# Single‐Cell and Spatial Transcriptomics Delineate the Microstructure and Immune Landscape of Intrahepatic Cholangiocarcinoma in the Leading‐Edge Area

**DOI:** 10.1002/advs.202412740

**Published:** 2024-12-24

**Authors:** Li Zuyin, Li Zhao, Cheng Qian, Zhang Changkun, Ma Delin, Hao Jialing, Chen Zhuomiaoyu, Li Yuzi, Zheng Jiaxi, Gao Jie, Zhu Jiye

**Affiliations:** ^1^ Department of Hepatobiliary Surgery Peking University Organ Transplantation Institute Peking University People's Hospital Beijing 100044 China; ^2^ Beijing Key Surgical Basic Research Laboratory of Liver Cirrhosis and Liver Cancer Beijing 100044 China

**Keywords:** immune landscape, intrahepatic cholangiocarcinoma, leading‐edge area, spatial transcriptomics

## Abstract

Intrahepatic cholangiocarcinoma (ICC) tumor cells and their interactions with the immune microenvironment, particularly at the leading‐edge area, have been underexplored. This study employs single‐cell RNA sequencing (scRNA‐seq) and spatial transcriptome (ST) analysis on samples from the tumor core, adjacent non‐tumorous tissue, and the leading‐edge area of nine ICC patients. These findings indicate that tumor cells at the leading‐edge area demonstrate enhanced proliferation and are tightly associated with the stroma, including endothelial cells and POSTN+ FAP+ fibroblasts. Notably, CD8+ T cells in this region exhibit a naive phenotype with low cytotoxicity and signs of exhaustion, likely due to compromised antigen presentation by antigen‐presenting cells (APCs). The predominant CD8+ T cell subset, mucosal‐associated invariant T (MAIT) cells, recruits SPP1+ macrophages within the stroma. This interaction, along with the presence of POSTN+ cancer‐associated fibroblasts (CAFs) and endothelial cells, forms a unique “triad structure” that fosters tumor growth and ICC progression. The research highlights the intricate characteristics and interactions of ICC tumor cells in the leading‐edge area, offering insights into potential therapeutic targets for intervention.

## Graphical abstract

This study employs single‐cell RNA sequencing and spatial transcriptomics to delineate the microstructure and immune landscape of Intrahepatic cholangiocarcinoma (ICC), particularly in the leading‐edge area. It reveals enhanced tumor cell proliferation, a unique immune environment, and a new defined microstructure called “triad structure,” that occurred in the ICC tumors’ leading edge.

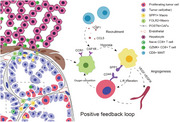

## 1 Introduction

Intrahepatic cholangiocarcinoma (ICC) ranks as the second leading type of primary liver malignancy, noted for its exceptionally aggressive essence and a generally poor prognosis.^[^ ^1^ ^,^ ^2^ ^]^ Most patients have limited treatment chances for complete surgical resection when they are often first diagnosed at an advanced stage, and there is still a high risk of recurrence after surgery.^[^ ^3^ ^]^ At present, new treatment methods, such as targeted therapy (inhibitors or mutation‐targeting agents) and immunotherapy, become the more promising approaches for late‐stage patients.^[^ ^4^ ^]^ Targeted therapy has achieved promising results through targeting specific genetic mutations in patients. Targeted drug therapy, such as FGFR2/IDH‐1 inhibitors, is used in subgroups of patients with specific mutational alterations and has achieved good clinical outcomes.^[^ ^5^, ^6^, ^7^, ^8^ ^]^ Patient responses to identical treatment modalities can diverge significantly, a phenomenon particularly pronounced in the realms of targeted and immunotherapies. This underscores the imperative to explore the intricacies of the tumor microenvironment (TME), aiming to achieve a more nuanced understanding of its characteristics.

In the quest to enhance our grasp of the TME in ICC, the advent of scRNA‐seq and ST technologies has bestowed upon researchers a suite of potent and exacting investigative instruments. The combination of two technologies enables researchers to explore the complex cellular interactions and the spatial relationships that define the intricate network within the tumor's ecosystem, which also enables us to focus on a long‐overlooked area, the leading‐edge area of ICC tumor, also known as the tumor‐normal interface or invasive tumor front. We and others have delved into the heterogeneity of ICC tumors, uncovering that the immune microenvironment exhibits significant variation across different regions within the same tumor.^[^ ^9^ ^]^ This discovery prompted our subsequent inquiry into the unique phenomena occurring in a particularly critical area—the leading‐edge zone of the ICC tumor.

In prior studies, Zhou and colleagues pinpointed a 500‐µm‐wide band encircling the periphery of liver cancer tumors, naming it “the invasive zone.” Within this region, the emission of Serum Amyloid A proteins (SAAs) by compromised hepatocytes was found to summon macrophages and incite their M2 polarization. This process is believed to foster a localized state of immunosuppression, which could, in turn, fuel the advancement of the tumor.^[^ ^10^ ^]^ Meanwhile, Li's research shed light on the significant aggregation of renal cell carcinoma (RCC) cells at the invasive tumor front, characterized by a pronounced epithelial‐mesenchymal transition (EMT).^[^ ^11^ ^]^ Bouchard et al. found the crucial interplay between tumor and stromal cells, showing that their communication within the tumor microenvironment activates distinct biological pathways based on location—edge or core. They emphasized phenotypic distinctions in tumor cells when cultivated with fibroblasts sourced from the tumor's invasive edge versus its core.^[^ ^12^ ^]^

The collective evidence points to the tumor's leading edge, or the tumor‐normal interface, as a region that is intimately associated with the tumor's specific behaviors and progression. Given the heightened malignancy observed in ICC, our study undertook an in‐depth single‐cell and spatial transcriptome analysis on a cohort of nine ICC patients. This research was designed to reveal the characteristics of the TME in tumor's leading edge, encompassing the distribution of stromal and immune cells, the tumor niche's surrounding ecosystem, and the complex interplay among diverse cell types. The findings from this study provide novel and profound insights into the intricate dynamics of the ICC ecosystem, suggesting potential therapeutic targets that may be instrumental in impeding tumor advancement.

## 2 Results

### 2.1 The General Outline of the Leading‐Edge Area Depicted by Single‐Cell and Spatial Transcriptome Analysis

**Figure 1:**
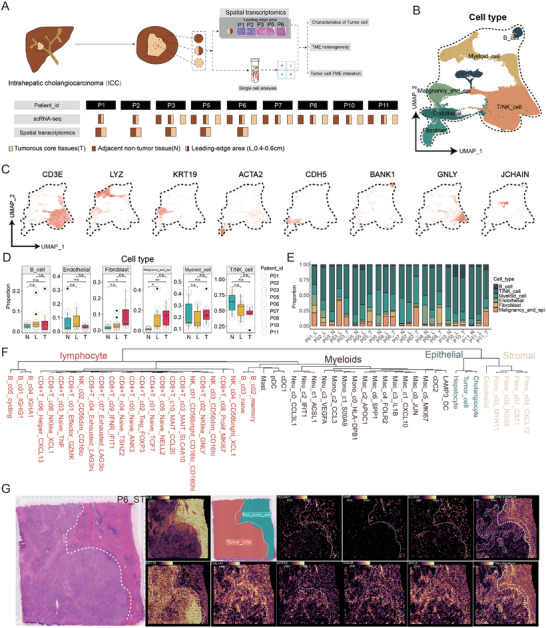
The general outline of the leading‐edge area depicted by single‐cell and spatial transcriptome analysis. A) Schematic diagram of the entire project, including patient‐matched sequencing information. T: tumorous core tissues, N: matched non‐tumor tissue (>3 cm from the border), L: leading‐edge area (expand ≈0.5 cm to both sides from the borderline). B) The UMAP visualization of the distribution of predominant cell populations, each distinguished by its specific color coding. C) UMAP plots illustrate the expression levels of key genes across diverse cell types. D) The distribution of major cell types across the indicated groups. E) Proportion of all cell types within the indicated samples. F) The tree plot represents the transcriptomic similarity of cell subtypes of four major classical cell types. G) Spatial transcriptomic results of the P6 patient, including H&E staining of tumor tissue, tumor‐side marker (KRT19), and non‐tumor side marker (ALB), and the distribution of fibroblast (fibro) and endothelial (endo) signature and corresponding markers (Fibroblast: COL1A1, ACTA2, TAGLN, and MYL9; endothelial: PECAM1, VWF, and CLDN5). *p < 0.05, **p < 0.01, ***p < 0.001. No significant difference (n.s.).

To fully elucidate the features of the leading‐edge area in ICC, we amassed tissue samples from nine ICC patients. This collection included core tumor tissues (T, with seven samples), samples from the leading‐edge area (L, with nine samples), and corresponding non‐neoplastic adjacent tissues (N, with nine samples), all of which were subjected to single‐cell RNA sequencing (scRNA‐seq). Furthermore, well‐preserved leading‐edge area samples (*n* = 5) underwent spatial transcriptomics (ST) sequencing on the 10x Genomics Visium platform (**Figure** **1**A; Figure S1A, Supporting Information). Comprehensive clinical profiles and pathological evidence for the ICC patients enrolled in this study are detailed in Table S1 and Figure S1B (Supporting Information), respectively.

In summary, ≈230 000 high‐quality single‐cell transcriptomes were retained after quality control assessments. Across the patient cohort, we discerned six predominant cell types, including myeloid cells (LYZ), epithelial (including malignant tumor cells, hepatocytes, and cholangiocytes) (KRT19), fibroblasts (ACTA2), endothelial cells (CDH5), T/NK cell (CD3E, GNLY), and B cells (BANK1, JCHAIN), based on their canonical markers (Figure 1B,C).

Diverse primary subpopulations are present across various tumor locales (Figure 1D) and within each sample (Figure 1E). However, these subpopulations exhibit varying proportions, thus highlighting the regional, as well as the inter‐ and intra‐patient heterogeneity within the tumor immune microenvironment in ICCs. In agreement with other studies,^[^ ^13^ ^]^ our study revealed a marked enrichment of fibroblasts within the tumor tissues (Figure 1D). Apart from tumor cells, other major immune cells may not show much difference in distribution tendency, implying the difference in different regions of ICC tumors may need more subtle investigation and the refined cluster of major cell types. Therefore, we here have summarized all cell subpopulations identified and depicted in this study, and the “hclust” algorithm was used to demonstrate the transcriptomic similarity among cell subpopulations (Figure 1F).

To depict the characteristics of ICC spatial organization in the leading‐edge (L) area, we used ALB and KRT19 to define the tumor side and non‐tumor side on the L‐area slice (Figure 1G, left, and Figure S1C, Supporting Information). Meanwhile, we used the endothelial and fibroblast signature derived from matched scRNA data of the L area to outline the stromal area in each slice (Figure 1G, right, and Figure S1C, Supporting Information). The corresponding canonical markers of endothelial cells (PECAM1, VWF, and CLDN5) and fibroblasts (COL1A1, ACTA2, TAGLN, and MYL9) also prove the feasibility of this strategy. In line with the previous study,^[^ ^14^ ^]^ the stromal region acts as a barrier at the tumor‐normal interface and also extends into the tumor region, dispersing or encircling the tumor cells. This observation underscores the complex interplay and spatial distribution of stromal elements with tumor cells.

### 2.2 ScRNA Sequencing and Spatial Transcriptomics Reveal ICC Tumor Cell Heterogeneity at the Invasive Tumor Front

**Figure 2:**
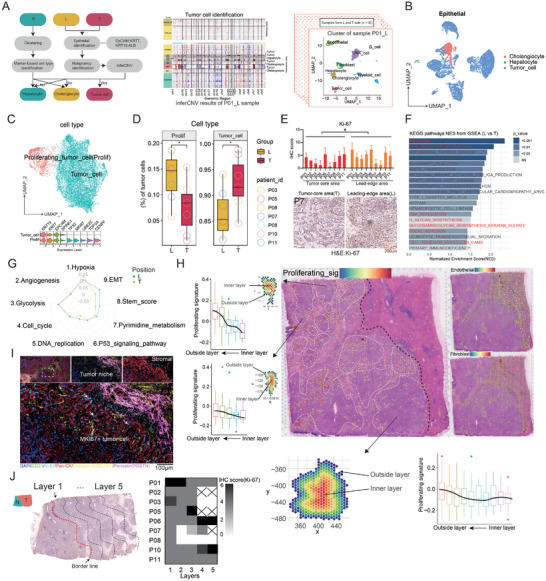
ScRNA sequencing and spatial transcriptomics reveal ICC tumor cell heterogeneity at the leading‐edge area. A) Schematic diagram of the strategy to identify tumor cells. B) UMAP plots of three epithelial subtypes (Cholangiocyte, hepatocyte, and tumor cell) were identified using inferCNV and gene marker‐based strategy. C) UMAP plots of tumor cell subtypes (top) and their corresponding markers as demonstrated by violin plot (bottom). D) The proportion of proliferating tumor cells in the lead‐edge and core regions of ICC tumor. E) Immunohistochemistry (IHC) staining images (bottom) and statistic differences (top) of Ki‐67+ tumor cells in the L‐ and T‐side regions within ICC samples. F) KEGG pathway enrichment analysis was applied to examine the differentially expressed genes (DEGs) between tumor cells from the L and T sides. G) The “addmodulescore” algorithm was used to score the signature of nine biological processes in tumor cells from L‐side and T‐side ICC samples. H) The spatial dot plots showed the distribution of proliferating tumor cells and stromal (fibroblast and endothelial) and the tumor niches within were sliced by layers and calculated the score of proliferating signature. I) The mIHC results of ICC tumors in the leading‐edge zone show the distribution relationship between proliferative tumor cells (Pan‐ck+, Ki‐67+) and the stromal area [composed of endothelial cells (CD31+) and fibroblasts (collagen I+, periostin+)]. J) The invasive tumor front was sliced by layers (left) and the IHC scores (right) were calculated. *p < 0.05, **p < 0.01, ***p < 0.001. No significant difference (n.s.).

To evaluate the spatially characteristic disparities of tumor cells within ICC, we first applied the inferCNV algorithm which used immune cells as a reference, together with a marker‐based strategy, to identify tumor cells from epithelial (**Figure** **2**A,B). All patients contain cholangiocytes and ICC tumor cells, but a small number of patients (P5, P6, P7) lack hepatocytes based on our single‐cell dissociation strategy (Figure S2A,B, Supporting Information). We found that tumor cell similarity is more obvious in intra‐patient samples than in inter‐patient samples (Figure S2C, Supporting Information). And those previously reported genes such as CFTR, TM4SF4, and FXYD2, which characterize bile duct cells or cholangiocytes, have also been well reproduced in our single‐cell data (Figure S2D, Supporting Information). We extracted and re‐clustered ICC tumor cells and named those overexpressing MKI67, TOP2A, and UBE2C as proliferating tumor cells (Prolif) (Figure 2C). We found that proliferating tumor cells were more enriched in the leading‐edge area (L) compared with the tumor‐core area (T) (Figure 2D; Figure S2E–H, Supporting Information), which is further proved by immunohistochemistry (IHC) evidence (Figure 2E). Further, we investigated the potential driver transcription factors (TFs) using SCENIC and found that E2F1 and CEBPB (Figure S2I, Supporting Information), associated with proliferation and stemness, were significantly upregulated in L‐side tumor cells, which further proved our above finding. We then collected a set of differentially expressed genes (DEGs) resulting from the comparative analysis of tumor cells from the L side and T side, conducting a KEGG pathway enrichment analysis. We found that in addition to the above‐mentioned represented proliferating characteristic (e.g., DNA replication), tumor cells from the leading‐edge area are also enriched in pathways like ribosome, ECM receptor interaction, and cell adhesion molecules (CAMs), etc (Figure 2F). We used the “addmodulescore” algorithm to evaluate other common behaviors of tumor cells and found that the tumor cells derived from the tumor‐core area exhibit obvious hypoxia, while the tumor cells in the leading‐edge region have significant advantages in stemness and EMT behaviors (Figure 2G). We also found that stemness‐related genes, e.g., CD44 and CD133, were overexpressed in L‐side tumor cells, and overexpression of CD44 brought a worse prognosis in the FDU‐ICC cohort (Figure S2J,K, Supporting Information).

To observe the distribution of proliferating tumor cells in ICC tissues, we constructed a signature of proliferative markers and enriched them in the ST data. We found that proliferating tumor cells were mainly distributed around the tumor niche (or cancer cell aggregates), close to the stromal area which is composed of endothelial and fibroblast (Figure 2H; Figure S2L, Supporting Information). We circled out the entire tumor niche and performed onion‐like stratification. The proliferative signal of the inner‐layer tumor cells is weaker than that of the outer‐layer tumor cells (Figure 2H). Using multiplex immunohistochemistry (mIHC) technology, we confirmed that the location of proliferating tumor cells (Ki‐67+, Pan‐CK+) is close to where endothelial cells (CD31+) and fibroblasts (Collagen I+) are found (Figure 2I). However, in the leading‐edge area, as tumor cells approach the normal‐tumor interface line, it seems that they do not necessarily proliferate faster (Figure 2J).

### 2.3 Naive CD8+ T Cells Dominate and MAIT Cells Infiltrate the Leading‐Edge Area

**Figure 3:**
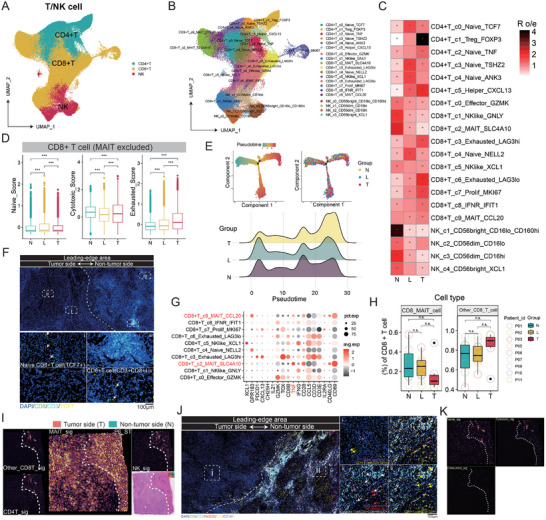
Naive CD8+ T cells dominate and MAIT cells infiltrate in the leading‐edge area. A,B) UMAP plots show the major T/NK cluster (A) and their cell subsets (B). C) The distribution preference of CD8+ T cell subpopulations at different regions. D) The naïve, cytotoxic, and exhausted score of CD8+ T cell (CD8+ MAIT excluded) at different positions. E) Pseudo‐time trajectory analysis examines the evolution of CD8+ T cells at various pseudo‐time points and positions (top), alongside their distribution across the pseudo‐time spectrum (bottom). F) The multicolor immunofluorescence images show that naive CD8+ T cells (CD8+, TCF+) were detected in the tumor side of the leading‐edge zone, while the majority of CD8+ T cells are located in the non‐tumor area. G) Dot plot shows the expression of effector markers across CD8+ cell subsets. H) The distribution of MAIT cells in different regions. I) Spatial dot plots depict the arrangement of CD8+ T cells, MAIT cells, CD4+ T cells, and NK cells within the tumor's leading‐edge region. J) The mIHC images confirmed that only a small number of MAIT cells and CD4+ T cells existed in the tumor side of the leading‐edge area, while the majority of CD4+ T cells, NK cells, and cytotoxic CD8+ T cells were on the non‐tumor side. K) The spatial dot plots show the distribution of naïve‐, cytotoxic‐, and exhausted‐enriched spots calculated by the “addmodulescore” algorithm. *p < 0.05, **p < 0.01, ***p < 0.001. No significant difference (n.s.).

T/NK cells play a pivotal role in the TME. To delve deeper into the characteristics of T/NK cells in the leading‐edge zone, we conducted a refined clustering analysis, categorizing the T/NK cells—comprising CD4+ T cells, CD8+ T cells, and NK cells—into 20 distinct clusters. (**Figure** **3**A,B; Figure S3A, Supporting Information). CD8+ T cell subsets include the effector T cells (c0_Effector_GZMK), two types of mucosal‐associated invariant T (MAIT) cells (c2_MAIT_SLC4A10 and c9_MAIT_CCL20), one naïve subset (c4_Naive_NELL2) and two natural killer cell‐like subsets(c2_NKlike_XCL1 and c1_NKlike_GNLY), one interferon responded (IFNR) T cells(c8_IFNR_IFIT1), and two types of exhausted T cells (c3 and c6) and a proliferating subset (c7_Prolif_MKI67). CD4+ T cell (six clusters) and NK cell (four clusters) were also subclustered according to their canonical markers (Figure S3A, Supporting Information). Our findings indicate that both CD8+ and CD4+ naïve T cells are notably enriched in the leading‐edge area of the tumor. In contrast, Treg cells and exhausted CD8+ T cells exhibit a propensity for localization within the tumor‐core region (Figure 3C). Upon scrutinizing the signature genes as previously characterized,^[^ ^15^ ^]^ we discerned that CD8+ T cells predominantly exhibit a naïve phenotype, with diminished cytotoxic potential and a low level of exhaustion in the L‐side area (Figure 3D). These evaluated CD8+ T cells did not include CD8+ MAIT cells due to the different anti‐tumor modes of these two types of CD8+ T cells. Pseudotime analysis constructed the trajectory of CD8+ T cell maturation from a naive state (Figure S3B–D, Supporting Information). We observed that the majority of CD8+ T cells from the leading‐edge area were positioned at the onset of pseudo‐time, whereas those from the tumor‐core area were predominantly found at the terminus of pseudo‐time. This distribution suggests distinct states of CD8+ T cells across different tumor regions (Figure 3E). The results of mIHC also observed that there were a few naïve CD8+ T cells i) in the tumor side of the leading‐edge area, and CD8+ T cells were scarce ii), with most of them located on the non‐tumor side iii, iv) (Figure 3F). Unlike other classic CD8+ T cells, which rely on the cytotoxic molecule Granzyme K (GZMK), CD8+ MAIT cells primarily exert their anti‐tumor effects through the non‐specific molecules, tumor necrosis factor (TNF) (Figure 3G). We also noticed a tendency for CD8+ MAIT cells to be enriched in the tumor side of the leading‐edge area, although the difference was not statistically significant (Figure 3H). ST and mIHC results proved that CD8+T cells, CD4+T cells, and NK cells are mostly located on the non‐tumor side of the frontier region, while MAIT infiltrates into the tumor side (Figure 3I,J), suggesting the initial anti‐tumor role of MAIT in the leading‐edge area. The expression of MAIT effector molecules (TNF) and GZMB, the marker of cytotoxic CD8+T cells (Figure S3E, Supporting Information), as well as the signature of naïve, cytotoxic, and exhausted activities (Figure 3K), also prove the above findings. However, for the entire CD8+ cell subpopulation, the cytotoxic effect of MAIT is not outstanding (Figure S3F, Supporting Information), unlike those effector (c0), exhausted (c3), or NK‐like CD8+ T cells (c1 and c5), and the occurrence of MAIT cells suggest they serve as the vanguard of anti‐tumor CD8+ cells in this region. Meanwhile, we noticed that CD8+ MAIT cells exhibit stronger expression of anti‐tumor molecules (IFNG and TNF) in the normal liver tissue and exhibit a gradual decrease in anti‐tumor ability from normal liver tissue to the invasive tumor front, and to core tissue (Figure S3G, Supporting Information), which might be associated with the metabolic change in different regions, noticing the elevated levels of hypoxia and glycolysis activities (Figure S3H, Supporting Information). While in tumor‐core tissues, canonical CD8+T cells with GZMK/B/A and exhausted molecules (TOX and PDCD1) dominate (Figure S3I, Supporting Information), which is completely different from the naïve and low‐cytotoxic/exhausted states of CD8+T cells in the leading‐edge area.

It is worth noting that this phenomenon of naive cells accumulating in the tumor‐normal interface not only occurs in CD4+ and CD8+T cells but also in B cells (Figure S4A,B, Supporting Information). We found that naïve (B_c03_naive) and memory B cells (B_c02_memory) enriched in the leading‐edge region, while tumor‐core tissue is more abundant with B cells that produce antibodies (B_c01_IGHG1) (Figure S4C, Supporting Information). Surprisingly, Breg‐related genes were found to be enriched in the non‐tumoral tissue (N), with a small portion enriched in the L area (Figure S4D, Supporting Information). This may be associated with the distribution of a greater number of tertiary lymphoid structures (TLS) in the non‐tumoral region (data not shown). And B cells infiltrated into tumor‐core tissues acquired other phenotypes like angiogenesis, or colonization ability due to environment shift (Figure S4E, Supporting Information).

Together, these above findings suggest the immature state of lymphocytes in the leading‐edge area and the behavior of lymphocytes against tumor cells may be more intense in the tumor‐core area, whether using antibodies or cytotoxic molecules.

### 2.4 The Antigen‐Presenting Capacity of APCs in the Leading‐Edge Area is Impaired

**Figure 4:**
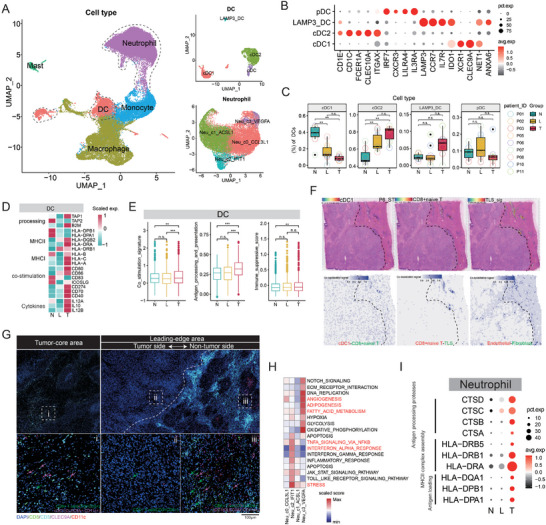
The antigen‐presenting capacity of APCs in the leading‐edge area is impaired. A) The UMAP visualization of myeloid cells subsets (left) and subsets of DC and neutrophils (right). B) The circular plot delineates the expression profiles of signature genes across different DC subsets. C) Distribution of DC subsets at different locations. D) Heatmap shows the relative levels of antigen‐presentation‐related genes at different regions. E) The comparison of three different biological processes of DC at different locations. F) The spatial dot plots show the relative distribution of cDC1, CD8+ naive T cell, and TLS and the levels of their colocalization signal. G) The mIHC images revealed the quantity and positional relationship of CD8+ T cells and cDC1 in the leading edge and core area of the ICC tumor. H) The results of GSEA enrichment of gene sets in all neutrophil subsets. I) The circular plot illustrates the gene expression levels associated with antigen presentation processes, categorized by location. *p < 0.05, **p < 0.01, ***p < 0.001. No significant difference (n.s.).

To delve into the function of myeloid cells at the leading‐edge area, we applied dimensionality reduction and unsupervised clustering to all myeloid cells (**Figure** **4**A). This chapter mainly discussed two main subclusters, including dendritic cells (DCs) and neutrophils. Among the DCs, we distinguished cDC1 (XCR1 and CLEC9A), cDC2 (CLEC10A, and FCER1A), LAMP3 DC (LAMP3 and CCR7), and pDC (IRF7 and IL3RA) (Figure 4B). We found that cDC2 was mostly enriched in the leading‐edge and tumor‐core area, and cDC1, however, shows the opposite condition (Figure 4C). DC is one of the classic antigen‐presenting cells (APCs). However, we found that the antigen presentation ability and co‐simulation activity of DCs were significantly reduced in the leading‐edge (L) area compared to the tumor‐core (T) region (Figure 4D,E). Our above results indicate that CD8+ T cells in the leading‐edge area exhibit a naïve, low‐cytotoxic phenotype. It is worth noting that naïve CD8+ T cell maturation requires antigen presentation with cDC1 and their proximity in location to occur a co‐stimulation event.^[^ ^16^ ^]^ However, in our ICC samples, cDC1 rarely infiltrates the tumor side of the leading‐edge area and mostly aggregates in the tertiary lymphoid structures (TLS) of the non‐tumor area, so as CD8+ naïve T cells (Figure 4F; Figure S5A, Supporting Information). The results of mIHC also proved that there were only a few CD8+ T cells on the tumor side of the L area, and almost no cDC1 cells were found ii), compared to the tumor‐core area i) and the non‐tumor side iii) of the L area (Figure 4G). The above finding suggesting the immaturity of CD8+ T cells might be associated with the incompetent antigen presentation of DCs.

Despite DCs, our data also identified 4 clusters of neutrophil subsets (Figure 4A; Figure S5B, Supporting Information). Neutrophils of cluster 3 (Neu_C3_VEGFA, CD74+/HLA‐DRA+) displayed a mixed phenotype with angiogenesis, lipid metabolism‐related, and antigen‐presentation characteristics and they enriched in both the leading‐edge and tumor‐core region (Figure S5B–D, Supporting Information; Figure 4H). However, whether in the major neutrophil group or the subgroup (Neu_C3_VEGFA), their antigen‐presenting function in the leading‐edge area seems to be deficient (Figure 4I; Figure S5E, Supporting Information). This is consistent with the phenomenon observed on DCs. It is worth noting that this subpopulation (Neu_C3_VEGFA) may represent a potentially harmful subgroup, similar to the SPP1+ macrophages that we will discuss next. Enrichment analysis of its genesets in bulk RNA‐seq data of FDU‐ICC cohort suggests that it is a high‐risk factor for patient mortality (Figure S5F, Supporting Information). We also noticed that the interferon‐responded and cell stress‐related cluster (Neu_c2_IFIT1) (Figure 4H) is mostly enriched in the leading‐edge area (Figure S5C, Supporting Information). Unlike cluster 3 (Neu_c3_VEGFA), transcription factor regulons enriched in c2_IFIT1 neutrophils were mostly cell stress‐related (IRF9, IRF7, and STAT2) (Figure S5G, Supporting Information), this may be related to the stress state generated by this subgroup after receiving interferon signals.

### 2.5 Pro‐Angiogenic SPP1+ Macrophages are the Main Macrophage Subsets that Infiltrate into the Tumor Side of the Leading‐Edge Area

**Figure 5:**
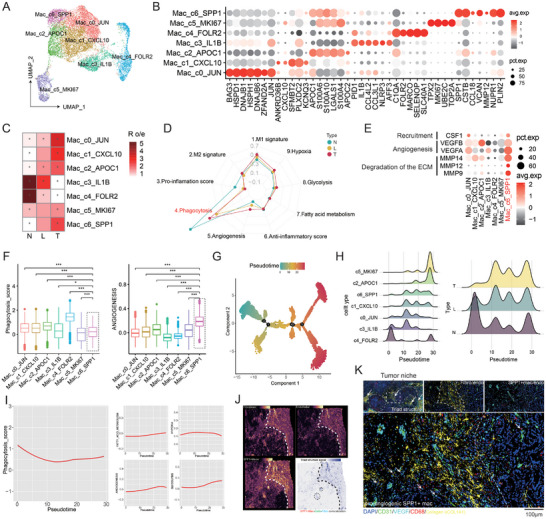
Pro‐angiogenic SPP1+ macrophages are the main macrophage subsets that infiltrate into the tumor side of the leading‐edge area. A) The UMAP plot delineates distinct subsets of macrophages, with each cell type demarcated by a unique color. B) The circular plot reveals the marker gene expression of each macrophage subset. C) The preferential distribution patterns of macrophage subpopulations across distinct anatomical regions. D) The comparison of nine different biological processes of macrophages among different regions. E) The expression of specific functional genes among different macrophage subsets. F) The comparison of phagocytic and angiogenic functions among macrophage subpopulations. G,H) Pseudo‐time analysis of macrophages (G) and distribution of various macrophage subpopulations (H, left) and macrophages at different locations (H, right) over pseudo‐time. I) The changes in five different cellular processes of macrophages over pseudo‐time. J) Spatial dot plot shows the distribution of SPP1+macrophage, fibroblast, and endothelial and corresponding tricellular colocalization. K) The mIHC results prove the presence of pro‐angiogenic SPP1+ macrophages (CD68+ VEGF+) within the Triad structure. *p < 0.05, **p < 0.01, ***p < 0.001. No significant difference (n.s.).

Macrophages are also an important component of anti‐tumor immunity, and their functions in the immune microenvironment are more versatile. Antigen presentation ability is also one of their abilities. However, in our ICC cohort, like many other myeloid cells, their antigen presentation and processing ability are also weaker in the leading edge compared to the core area (Figure S6A, Supporting Information). To further investigate the regional heterogeneity of macrophages, we further refined macrophage subgroups (**Figure** **5**A,B), including the previously reported FOLR2+ macrophages (c4),^[^ ^17^, ^18^ ^]^ the transitional state IL1B+ subsets (c3), the cell stress‐related JUN+ subsets (c0), and the previously considered harmful SPP1+ subgroup (c6).^[^ ^19^ ^]^ In terms of distribution, SPP1+ cluster (c6), APOC1+ cluster (c2), and JUN+ cluster (c0) show gradual enrichment from the invasive tumor front to the core region, while those previously deemed “good” subsets, like FOLR2+ (c4) and IL1B+ (c3) cluster, preferentially accumulated in the non‐tumor area (Figure 5C). We found that overall, macrophages have the worst phagocytic ability in the frontier area of invasion, while macrophages in the core area of the ICC tumor exhibit a hypoxic phenotype, which enriched in pathways such as hypoxia and glycolysis, consistent with the phenomenon observed in tumor cells (Figure 5D). Among these subsets of macrophages, Mac_c06_SPP1 (SPP1+, PLIN2+) exhibits a distinct malignant phenotype, manifested in its high expression of the degradation of the extracellular matrix (ECM)‐related genes (MMP14, MMP12, and MMP9), enhanced ability to promote angiogenesis, and weakened ability to phagocytose tumor cells (Figure 5E,F). We inferred developmental trajectories of macrophages in these subsets (Figure 5G; Figure S6B, Supporting Information) and found that FOLR2+ macrophages(c4) gradually developed toward SPP1+ macrophages (c6) and finally to MKI67+ subsets (c5) (Figure 5H), accompanying the faded ability of phagocytosis and enhanced fatty acid metabolism, hypoxia‐ and glycolysis‐related process (Figure 5I). A diverse array of TFs was observed to be activated across the varied macrophage subpopulations. Notably, TFs associated with fatty acid metabolism, particularly PPARG, were selectively activated in the SPP1+ macrophages (Figure S6C, Supporting Information). This activation pattern implies a connection between the emergence of the SPP1+ subset and the metabolic pathways centered on fatty acid processing.

To better depict the distribution of each macrophage subset, we performed spatial transcriptome projection on the markers of each subset (Figure S6D, Supporting Information), and we noticed an irony phenomenon that the FOLR2+ subgroup with the intact phagocytic function (Figure 5F) was mainly located in the non‐tumor side of the invasive tumor front (Figure S6E, Supporting Information), while the SPP1+ subgroup, which was the worst of that function, was mainly distributed in the tumor side, more specifically in the stromal region, composed of endothelial cells and fibroblasts (Figure 5J,K; Figure S6F, Supporting Information), which we termed “triad structure.” As previously described, this triad structure forms an immune barrier that hinders T cells from infiltrating into the tumor side in HCC and CRC,^[^ ^14^ ^]^ and this phenomenon was also seen in ICC tumors (Figure 3I,J; Figure S6G, Supporting Information). In addition to the distribution of this structure at the tumor‐normal interface, we also found that this structure extends to the tumor side, scattering around the tumor niches (Figure 5K). Considering the proximity relationship between the SPP1+ subgroup and endothelial cells, through ligand‐receptor interaction analysis, which was based on the “Cellchat” algorithm, we found that it has a significant pro‐angiogenic function through VEGFA‐VEGFR1 interaction, and this phenomenon is more evident in the leading‐edge area (Figure S6H,I, Supporting Information).

### 2.6 POSTN+ FAP+ CAFs were the Major Fibroblast Subset that Composed the Triad Structure

**Figure 6:**
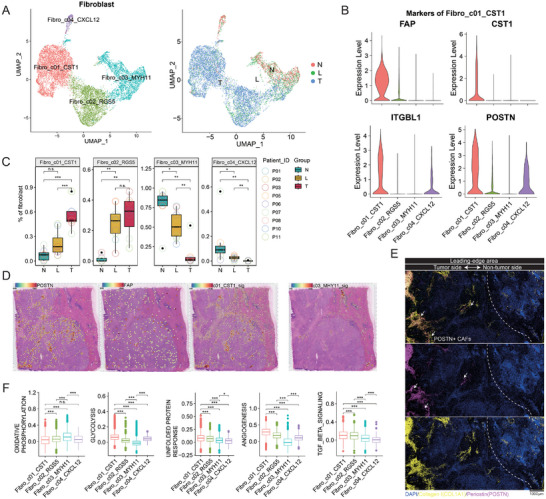
POSTN+ FAP+ CAFs were the major fibroblast subset that composed that triad structure. A) The UMAP visualization on the left delineates the distinct fibroblast subpopulations, while the corresponding plot on the right maps their distribution. B) The markers of CAFs in the c01 subgroup are highly expressed. C) The distribution of fibroblast subsets in different regions. D) The signature score of the c01 cluster and its markers enriched in the tumor side of the leading‐edge area, with the c03 cluster mainly located at the non‐tumor side. E) Collagen I‐producing fibroblasts are present on both sides of the leading‐edge area, while POSTN+ fibroblasts appear only on the tumor side. F) Analysis of pathway activity, quantified through the Gene Set Variation Analysis (GSVA), reveals comparative patterns among the diverse subtypes of fibroblasts, highlighting distinctive functional signatures. *p < 0.05, **p < 0.01, ***p < 0.001. No significant difference (n.s.).

Our above results indicate that SPP1+ macrophage subsets (Mac_c6_SPP1), endothelial cells, and fibroblasts have a co‐localization relationship and form a triad structure. We have not yet elucidated the functional characteristics of fibroblasts, since it was an important component of this structure. We then explored the fibroblasts of three aforementioned regions in our ICC cohort and identified 4 subclusters (**Figure** **6**A). The c03_MYH11 cluster was enriched with smooth muscle‐related genes, including MYH11 and TAGLN, suggesting it is a myofibroblast identity. The c02_RGS5 subset demonstrated heightened expression of pericyte markers, such as MCAM and RGS5. The c04_CXCL12 subgroup displayed characteristics of both antigen‐presenting cancer‐associated fibroblasts (apCAFs), marked by CD74 and HLA‐DRA, and immune‐related cancer‐associated fibroblasts (iCAFs), indicated by CXCL14 and CXCL12. This duality suggests that the c04_CXCL12 subgroup could be an immune‐modulatory subset (Figure S7A, Supporting Information). Meanwhile, the c01_CST1 subset was distinguished by elevated levels of FAP and POSTN expression, indicative of their identity as quintessential CAFs (Figure 6B). Notably, this FAP+ POSTN+ CAF(c01_CST1) is significantly enriched in the tumor side, while myofibroblasts (c03_MYH11) show less proportion in this area (Figure 6C). The results of ST also indicate that the marker genes of c01_CST1 subset, such as POSTN and FAP, are mainly expressed in the tumor side of the leading‐edge area, and so did the signature of this subset, which is composed of highly expressed genes calculated by FindAllMarkers algorithm, while the myofibroblast (c03_MYH11) subsets are mainly distributed in the non‐tumor side (Figure 6D). Our mIHC results also demonstrated that the fibroblast marker, Collagen I, is distributed on both sides of the L area, while POSTN+ CAFs are found only on the tumor side, corroborating our findings from single‐cell and spatial transcriptomics studies (Figure 6E). Among these four fibroblast subsets, a notable enrichment of gene sets linked to oxidative phosphorylation was identified within the myofibroblast subset (c03_MYH11), which is understandable, considering this subset mainly in the perivascular region. While the POSTN+FAP+ CAFs(c01_CST1) show enrichment in pathways like glycolysis, unfolded protein response, TGF‐beta signaling, and angiogenesis, suggesting that the environment in which it exists is related to hypoxia, and resulting corresponding countermeasures accordingly (Figure 6F). Together, the above findings show that POSTN+FAP+ CAFs were the major fibroblast subsets that composed that triad structure, not the myofibroblast or immune‐related subsets.

### 2.7 Cellular Crosstalk Between Tumor Cells and Immune Cells Shapes the Unique TME of the Invasive Tumor Front

**Figure 7:**
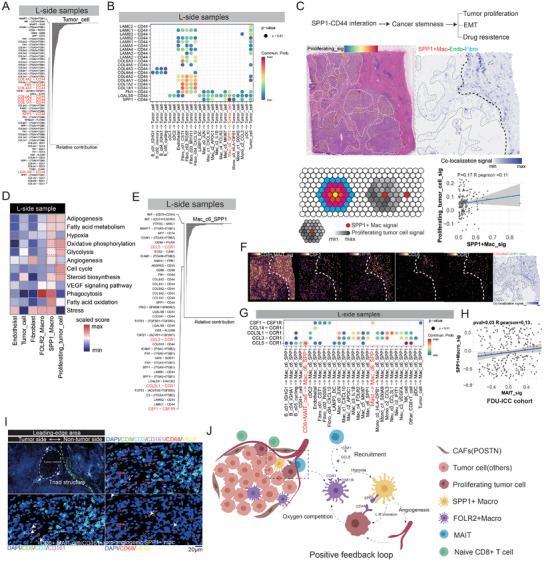
Cellular crosstalk between tumor cells and immune cells shapes the unique TME of the invasive tumor front. A) The L‐R contribution of tumor cells in the leading‐edge area. B) The CD44 receptor on tumor cells receives ligand sources from other cell subtypes. Circle dimensions correspond to the P values, and the likelihood of interaction is represented by color intensity. C) Schematic diagram of SPP1‐CD44 interaction promoting tumor cell stemness and altering tumor biological behavior (top). The relative positional relationship between proliferative tumor cells and triad signals (indicates the special stromal area, composed of endothelial, fibroblast, and SPP1+macrophages) (middle), as well as the correlation between the enriched SPP1+macrophage spots and the signal intensity of surrounding proliferating tumor cells (bottom). D) An assessment of differential pathway activities across cell subtypes within the leading edge, as determined by Gene Set Variation Analysis (GSVA). The scores of pathways are scaled across cell types. E) The L‐R contribution of SPP1+ macrophage in the leading‐edge region. F) The distribution of MAIT's signature on spatial slice, alongside the levels and spatial arrangement of ligand‐receptor interaction‐related genes (CSF1‐CSF1R, CCL5‐CCR1), as well as the co‐localization signaling intensity and distribution of MAIT with SPP1+macrophage. G) The specified interactions between SPP1+ macrophages and diverse cellular subtypes, mediated by L‐R pairs. H) The association between SPP1+ macrophage signature and MAIT cell signature within the bulk RNA‐sequencing dataset from Fudan University's ICC cohort. I) The mIHC images demonstrate the proximity of CD8+ MAIT cells to SPP1+ macrophages within the triad structure. J) Schematic diagram of intercellular interactions in the leading‐edge area.

To understand the cellular interactions that occurred in the invasive frontier region of ICC tumors. We first focused on tumor cells. Using the “cellchat” algorithm, we calculated the cellular interactions using tumor cells as receptors. We found that most of the receptors that interact with tumor cells are CD44 and ITGA families (**Figure** **7**A), which are related to the processes of tumor cell proliferation and invasive movement. Given the high proliferation of tumor cells in this area, we analyzed the cells that provide this ligand and found that SPP1+ macrophages (Mac_c6_SPP1) interact with tumor cells via the SPP1‐CD44 axis (Figure 7B), and this interaction was more intense at the leading edge (Figure S8A, Supporting Information). Prior research has established that the engagement of the SPP1‐CD44 axis is instrumental in fostering the stem‐like properties of tumor cells, which in turn enhances their proliferative capacity and induces epithelial‐mesenchymal transition (EMT) phenotypes (Figure 7C, top).^[^ ^20^ ^]^ This is consistent with the aforementioned characteristics of ICC tumor cells we have found in this region. Furthermore, our above investigations have identified that proliferative tumor cells predominantly reside in close proximity to the triad structure, which comprises SPP1‐expressing macrophages, endothelial cells, and POSTN+FAP+ fibroblasts (Figure 7C, middle). To elucidate the spatial relationship between SPP1+ macrophages and proliferating tumor cells (MKI67+), we screened for SPP1+ macrophage‐enriched spots on the ST slide, then calculated the surrounding signals of proliferative tumor cells, and finally determined the correlation between the two. The results indicate that the enriched spots of SPP1+ macrophage on the spatial transcriptome are indeed accompanied by enhanced proliferative tumor signals, and their distribution is spatially positively correlated, although weak (Figure 7C, bottom). We also found from the GEO database that similar experiments have been conducted, where co‐culturing tumor cells alone with monocyte‐derived macrophages (MDM, the initial state of macrophage, like FOLR2+ macrophage in our study) does not promote tumor growth. However, if exogenous human SPP1 protein is added, the expression of proliferation‐related genes (UBE2C, E2F1) in the co‐cultured tumor cells increases (Figure S8B, Supporting Information). The bulk RNA‐seq data from the ICC cohort at Fudan University corroborate the positive correlation between the enrichment of the SPP1+ macrophage signature and the elevated levels of both the tumor stemness signature and CD44 expression (Figure S8C,D, Supporting Information). The proximity relationship between SPP1+ macrophages and proliferating tumor cells has also been confirmed by mIHC technology (Figure S8E, Supporting Information). Notably, in ICC tumors, higher infiltrations of SPP1+ macrophage would incur a poor prognosis (Figure S8F, Supporting Information). Meanwhile, our above findings underscore the pivotal role of hypoxia in the genesis of SPP1+ macrophages (Figure 5I). Considering the distribution of relevant cells near SPP1+ macrophages, this hypoxia might be mostly caused by proliferating tumor cells due to their high proliferation and high oxygen‐consumption behaviors in the leading‐edge area (Figure 7D). Consequently, originally a good subgroup (FOLR2+ macrophage) was educated by the environment cellularly or metabolically and transformed to SPP1+ subsets, accompanied by fade‐away phagocytic ability, enhanced angiogenesis and the occurrence of bypass energy metabolism‐related processes (fatty acid oxidation and glycolysis) (Figure 7D). To confirm the scenario inferred by the results from single‐cell and spatial transcriptomics, we also performed in vitro experiment using the ICC cell line RBE. We observed that ICC tumor cells exhibited enhanced proliferation ability when stimulated with SPP1 protein and in the presence of fetal serum (mimicking the condition of tumor cells near blood vessels) (Figure S8G–J, Supporting Information). Additionally, after co‐culturing tumor cells with macrophages under limited oxygen conditions, we did observe an increase in VEGF expression in the macrophages (Figure S8K–M, Supporting Information).

Then we continued to consider how SPP1+ macrophages were recruited into the TME. Hence, we used SPP1+ macrophages as receptor cells to identify the top‐ranked interacting molecules (Figure 7E). Observing that CD8+ MAITs, rather than other CD8+ T cells or NK cells, infiltrate the tumor's leading edge, we identified a potential mechanism wherein CD8+ MAIT cells recruit SPP1+ macrophages into the TME via the CCL5‐CCR1 or CSF1‐CSF1R axis. Additionally, a co‐localization phenomenon with SPP1+ macrophages was observed (Figure 7F,G). In the ICC bulk RNA‐seq cohort of Fudan University, there exists a positive correlation between two signatures, SPP1+ macrophages and CD8+ MAIT cells (Figure 7H). The results of mIHC also confirmed the presence of SPP1+ macrophages around CD8+ MAIT cells in the triad structure of the leading‐edge area, as well as other types of macrophages (Figure 7I). This recruitment is notably enhanced at the leading edge (Figure S8N, Supporting Information). In addition, considering that SPP1+ macrophages were later educated and then formed, we also found that CD8+ MAIT also has a strong recruitment effect on the initial good subgroup (FOLR2+ subgroup) (Figure S8O, Supporting Information).

Therefore, here we have discovered and depicted an immune environment soil favorable for ICC tumor growth that appears in the leading‐edge zone. Here, the classic MHC‐I molecule‐dependent CD8+T cells scarcely infiltrate the tumor side, and show a naïve, low‐cytotoxicity phenotype, while the CD8+ MAITs with weak cytotoxic ability infiltrate it. CD8+ MAIT cells recruit a good subset of macrophages (FOLR2+) to infiltrate into the tumor microenvironment but are transformed to low‐phagocytosis SPP1+ subsets due to the oxygen competition with proliferating tumor cells nearby. Thus, a unique triad structure is formed, composed of SPP1+ macrophages, POSTN+ fibroblasts, and endothelial cells. Within this structure, POSTN+ fibroblasts and SPP1+ macrophages form a barrier that shields tumor cells from immune cell attacks. Endothelial cells provide nutrients to the tumor cells. The potential angiogenic effect of SPP1+ macrophage and its potential interaction with tumor cells (SPP1‐CD44) in the triad‐structure area leads to the formation of an environment favorable for ICC tumor growth in leading edge, thus forming a positive feedback loop (Figure 7J). It is worth noting that this phenomenon may not be limited to ICC tumors. In our subsequent studies, we observed a similar phenomenon in HCC (Figure S9A, arrow spotted, Supporting Information) as well, suggesting that this triad structure may be conserved across different tumors. This structure could potentially support tumor growth and serve as a potential therapeutic target.

## 3 Discussion

Our study, leveraging scRNA‐seq and ST technologies, has provided a comprehensive view of the characteristics of ICC tumor cells at the leading‐edge area and their orchestrated interactions with the triad structure that shape the immune microenvironment. Our findings underscore the enhanced proliferative capacity of tumor cells in the leading‐edge area, juxtaposed with a distribution that interfaces closely with stromal elements such as endothelial cells and POSTN+ fibroblasts.

Notably, the predominance of naïve CD8+ T cells and the infiltration of MAIT cells in this region suggest a unique immunological landscape that may influence tumor progression. It is noteworthy that in hepatocellular carcinoma (HCC), research by Wang and colleagues has identified an enrichment of double‐positive T cells (expressing both CD4+ and CD8+) in the leading‐edge zone. Specifically, this T cell subset displays hallmarks of activation and exhaustion, indicative of the robust anti‐tumor immune response occurring at the leading‐edge region of HCC.^[^ ^21^ ^]^ This observation contrasts with the scenario in ICC, where the leading‐edge zone appears to be characterized by an immune‐incompetent phenotype in our study. The naïve state of CD8+ T cells in the ICC leading‐edge area, characterized by low cytotoxicity and exhaustion, may be due to an impaired antigen‐presenting capacity of APCs, which is critical for T cell activation and differentiation. Our results also show the scarcity of cDC1 in the tumor side of the leading‐edge area. This observation is significant, as cDC1 is known to be crucial for initiating effective anti‐tumor immune responses.

Furthermore, the presence of SPP1+ macrophages in the triad structure, alongside endothelial and POSTN+ FAP+ CAFs, suggests a role in promoting tumor development through pro‐angiogenic properties and activation of CD44 in tumor cells. The spatial distribution of these macrophages and their correlation with proliferating tumor cells indicate a spatially coordinated interaction that may facilitate a supportive microenvironment for tumor growth. Our study also highlights the heterogeneity of macrophage subsets within the tumor microenvironment, with SPP1+ macrophages exhibiting a distinct phenotype associated with poor phagocytic ability and enhanced angiogenic potential. The transition from FOLR2+ macrophages to SPP1+ subsets may represent an adaptation to the microenvironmental stressors, such as hypoxia, which is prevalent in the leading‐edge area due to the high proliferative rate of tumor cells. It is worth noting that in liver cancer, researchers have proposed an onco‐fetal niche, which is similar to the triad structure we observed in ICC. The difference is that they have FOLR2+ tumor‐associated macrophage (TAM), PLVAP+ endothelial, and POSTN+CAF, and promote tumor EMT behavior in liver cancer.^[^ ^22^ ^]^ This may be related to the difference in blood supply richness between HCC and ICC tumors. Given that in clinical practice, although both occurred in the liver, we have clearly found that HCC has an out better blood supply than ICC, it is speculated that FOLR2+ TAM lacks this oxygen competition phenomenon with HCC tumor cells, without the phenotype transformation to SPP1+ macrophages. It also suggests that the triad structure composed of macrophages, fibroblasts, and endothelial cells exhibits different forms and functions in different types of cancer.

Despite the novel insights provided by our study, some limitations warrant consideration. The sample size is modest, which may constrain the generalizability of our findings. Additionally, while our study has concentrated on the interplay between tumor cells and finite immune cells, other cellular roles of TME, such as B cells and dendritic cells, may also play significant roles that require further investigation. Mast cells in our cohort may also have a potential recruitment effect on SPP1+ macrophages (Figure 7G; Figure S8N, Supporting Information); however, due to their abundance, we cannot determine whether they play a major role, and it is possible that we have underestimated their function in tumor immunity. Future research directions should encompass the validation of our findings within a more extensive patient cohort to ascertain the universality of the observed patterns in ICC. Elucidating the intricate mechanisms underlying the crosstalk between tumor cells and immune cells, particularly the specific role of triad structure in tumor progression, will be critical. Finally, based on our discoveries, developing novel therapeutic strategies targeting SPP1+ macrophages or CD8+ MAIT cells could offer more effective treatment options for ICC patients.

In conclusion, our study offers new perspectives on the characteristics of ICC tumor cells in the leading‐edge area and the complex interplay with the immune microenvironment, providing potential targets for the interception of interaction‐based therapies within ICC.

## 4 Experimental Section

### Data Resources and Descriptions—Tissue Acquisition, Storage, and Sequencing

Treatment‐naive ICC specimens, including adjacent normal liver tissue, the leading‐edge area (previously defined by Wang^[^ ^21^ ^]^), and tumor core tissues, were collected from ICC patients with voluntary written consent, and with the sanction of the Peking University People's Hospital's medical ethics committee. These specimens were stored at Tissue Storage Solution (MACS, # 130‐100‐008), and then divided into three parts, which were used for single‐cell sequencing, spatial transcriptome sequencing, and paraffin embedding, respectively. A comprehensive compilation of patient details is presented in Table S1 (Supporting Information).

### Data Resources and Descriptions—scRNA Sequencing

Fresh samples of adjacent normal liver, the tumor's leading edge, and core tissues were rinsed with 4 °C PBS and dissected into fragments. These fragments were immersed in a 10 mL EDTA‐enriched solution and agitated for an hour at a temperature of 37 °C. Subsequently, the tissues were treated with a 10 mL DTT‐enriched PBS solution (65 mm DTT, with 10% FBS added) for a duration of 15 min at the same temperature, with continuous agitation. The EDTA and DTT were eliminated by rinsing the tissues with PBS twice. The cells were then strained through a 100‐micron filter, centrifuged, and rinsed twice with PBS. The resulting cell suspensions were prepared for scRNA‐seq, performed by LC‐Bio Technology (Hangzhou, China), following the standard operating procedure on the 10X Genomics Chromium platform.

### Data Resources and Descriptions—Samples Processed for Spatial Transcriptomics

Spatial transcriptomic libraries were crafted utilizing the 10x Genomics Visium Spatial Gene Expression Reagent Kit. The leading‐edge tissue samples were bisected, with the well‐preserved half being encased in OCT and preserved at −80 °C for subsequent analysis. The counterpart half was allocated for scRNA sequencing. Prior to executing the complete procedure, a tissue optimization test was conducted using the 10x Genomics Visium Spatial Tissue Optimization with cryosections from ICC patient tumors, accompanied by fluorescence signal visualization, which determined 24 min as the ideal permeabilization duration. Subsequently, tissue sections from a cohort of 5 ICC patients were prepared for spatial transcriptomic sequencing in accordance with the protocol provided by LC‐Bio Technology (Hangzhou, China).

### Data Resources and Descriptions—Spatial Genomics Data Preparation and Computational Processing

The FASTQ files were aligned to the GRCh38 human genome using the Space Ranger (v2.1.0) software from 10x Genomics, yielding a count matrix as per genome annotations. Spatial transcriptomics samples underwent quality control through the Loupe Browser, with the selection of undamaged spots devoid of tissue folds. Further quality checks, including gene count, mitochondrial read percentage, and ribosomal fraction, were unnecessary as all spots met the criteria.

Processed gene‐spot matrices were subsequently analyzed using the Seurat package in R (version 4.2.1). The “SCTtransform” function was applied for normalization across spots. Dimensionality reduction and clustering were conducted using principal components analysis (PCA), selecting the top 9–11 principal components. Signature scoring was executed with the AddModuleScore function in Seurat. The gene lists of particular signatures were generated from scRNA‐seq data and pathways from the “msigdbr” library, with details provided in Table S2 (Supporting Information). Spatial feature expression was visualized with Seurat's SpatialFeaturePlot function.

For a tumor niche circled by stroma, the Loupe Browser 8 was used to select this area and then use the Concaveman algorithm to stratify this area into the onion‐like layer. Then map the intensity of the ICC proliferating signature of each layer onto it.

To determine the spatial relationship between proliferating tumor cells and SPP1+ macrophages, single‐cell‐derived SPP1+ macrophage signatures were utilized and projected onto spatial transcriptional data. The average proliferative signal from tumor cells within the hexagonal zones surrounding areas of elevated SPP1+ macrophage signatures was then computed and analyzed each spot on the tumor side of the spatial transcriptome. By assessing the correlation between the signals of SPP1+ macrophages and those of proliferating tumor cells, the spatial interplay between these two cell types was deduced. This streamlined method efficiently elucidates the potential co‐distribution and interaction of SPP1+ macrophages with proliferating tumor cells.

### Data Resources and Descriptions—Co‐Localization Signal Calculation

For the assessment of co‐localization signals among two or three cell types, which were represented by genes, signatures, or distinct signal types, a tiered system was employed that categorizes signal intensities into quartiles (0–3). This methodological approach allows us to quantify the co‐localization by multiplying the signal intensities at identical spatial locations. Consequently, the co‐localization signal was minimal when only a single signal was detected. Conversely, the co‐localization signal reaches its peak when both signals were concurrently present and exhibit high intensities, indicative of a robust co‐occurrence. This analytical framework was equally applicable for scenarios involving tricellular co‐localization, enhancing the ability to discern complex cellular interactions within the tissue microenvironment.

### Data Resources and Descriptions—scRNA‐Seq Data Processing

The raw data was executed with the “Seurat” package in R (version 4.2.1 and 4.3.1). Cells exceeding 25% mitochondrial gene content were filtered out, along with genes detected in less than 20 cells and cells expressing less than 500 genes. Seurat's “FindVariableFeatures” function pinpointed the genes with the highest variability. Dimensionality reduction was achieved through Principal Component Analysis (PCA), and batch effects were mitigated using Seurat's CCA (canonical correlation analysis) method. UMAP clustering revealed six primary clusters, with cell type annotations determined by integrating known and differentially expressed marker genes.

Signature scores were derived by averaging the scaled and centered expression values of genes within each signature, as calculated by Seurat's “AddModuleScore” function. The cell type signature scores utilized are detailed in Table S2 (Supporting Information).

### Hematoxylin and Eosin (H&E) Staining

Tissues were preserved in 10% neutral‐buffered formalin and subjected to a series of ethanol treatments for dehydration, followed by paraffin embedding and sectioning into 5‐µm slices using standard histological techniques. H&E staining was applied to the thin sections for microscopic examination.

### Multiplex Immunohistochemistry (mIHC), Immunohistochemistry (IHC), and Quantitative Analysis

The process of mIHC was carried out following the Opal Kit guidelines from PerkinElmer. The list of primary antibodies and their dilutions is provided in Table S3 (Supporting Information). The procedure began with deparaffinization of the sections in xylene and subsequent rehydration through a graded ethanol series. Microwave antigen retrieval was performed in a heated citric acid buffer at pH 6.0 for 10 min. To quench endogenous peroxidase activity, 3% hydrogen peroxide was applied for 30 min, followed by a 30‐min blockade of nonspecific binding sites with goat serum. Incubation with primary antibodies took place for 1 h in a humidified box at room temperature, succeeded by the application of secondary antibodies conjugated to horseradish peroxidase. Fluorescein TSA Plus was used for target visualization at a dilution of 1:200. Between staining steps, slides were subjected to a second round of microwave antigen retrieval in a heated citric acid buffer to eliminate excess antibodies. Nuclear staining was completed with DAPI, and the sections were mounted using a fluorescent mounting medium. The slices were then scanned and imaged utilizing the PerkinElmer Vectra3 imaging system.

Immunohistochemistry (IHC) was conducted in accordance with the methods detailed in previous research.^[^ ^23^ ^]^ Pathologists, who were unaware of the patient outcomes, evaluated the immunoreactivity of proliferative markers (Ki‐67) through image analysis, focusing on both staining density and intensity. The scoring system was defined with specific criteria: a score of 1 was given for staining that was either 0–25% dense or showed negative intensity, 2 for 26–50% density or weak intensity, 3 for 51–75% density or moderate intensity, and 4 for 76–100% density or strong intensity. The overall semi‐quantitative score was derived by multiplying the density score with the intensity score, yielding a possible score range between 1 and 16.

### Developmental Trajectory Inference

Developmental trajectory analysis was performed using the Monocle2 (version 2.12.0) algorithm to explore the potential lineage progression within CD8+ T cells, excluding the CD8+ MAIT subset, and macrophages. For this analysis, the 2000 highly variable genes, as determined by the differentialGeneTest algorithm, were chosen to build the developmental trajectory map.

### Transcription Factor Regulon Analysis

To expedite the computational process, the examination of regulatory networks and regulon activities was facilitated by the pySCENIC tool.^[^ ^24^ ^]^ The expression matrix with cell type information was first transformed into “*.loom” profile. And then use Python (3.7.10) to run “pyscenic grn,” “pyscenic ctx,” and “pyscenic aucell” sequentially. The differential transcription factors among cell types were visualized in the R platform.

### Cancer Hallmarks and GSEA Analyses

Gene sets for analysis were sourced from the “msigdbr” library (http://software.broadinstitute.org/gsea/msigdb/). The scoring methodology for gene signatures adhered to a previously reported approach. Essentially, each cell's gene signature score was derived from averaging the scaled expressions of the genes within the signature. To detect significant shifts in gene expression between leading‐edge and core tumor cells, the “FindAllMarkers” algorithm (Seurat) was applied.

### Survival Analysis

Survival analysis was conducted utilizing the “survfit” function in R. The Cox proportional hazards model was employed to determine the Hazard Ratio (HR), with the 95% confidence interval (CI) being calculated. The Kaplan–Meier (K–M) method was applied to estimate survival functions using the survfit function. The ICC mRNA cohort from Fudan University was utilized to elucidate the correlation between SPP1+ macrophages and the characteristic genes or gene sets of related cells. This dataset can be accessed in the supplementary materials of the publication.^[^ ^25^ ^]^ Patients were stratified into two cohorts based on the median expression levels of specific genes—such as CD44—or based on the calculated scores of certain gene signatures, like the SPP1+ macrophage or stemness signatures. The two‐sided log‐rank test was then applied to statistically compare the K–M survival curves between these groups.

### InferCNV

The R package inferCNV (version 1.14.2), accessible at its GitHub repository (https://github.com/broadinstitute/inferCNV), was employed to distinguish malignant cells by deducing chromosomal copy number variations (CNVs) from gene expression profiles. Leveraging T/NK cells, B cells, and myeloid cells as representative comparators of normal cell populations, The epithelial with high CNVs and high epithelial markers were estimated to be malignant. A gene ordering file was crafted, grounded in the human GRCh38 genome assembly, which outlined the precise chromosomal coordinates—both the start and end positions—for each gene. This file was integral to the “gene_order_file” parameter required by the inferCNV algorithm. By inputting the raw count matrix and annotation file into inferCNV, a rigorous cutoff threshold of 0.1 was implemented to identify significant CNVs, thereby refining the precision of the analysis in the detection of tumor cells.

### Cell–Cell Interactions Analysis

Interactions between cells, as indicated by the expression patterns of established Ligand‐Receptor (L‐R) pairs across various cell types, were quantified using the CellChat tool (version 1.6.1), available on its GitHub repository (https://github.com/sqjin/CellChat).^[^ ^26^ ^]^ Briefly, CellChat was utilized with gene expression data to map overexpressed ligands and receptors onto a protein‐protein interaction network, identifying significant ligand‐receptor interactions. Probability assessment and permutation testing within CellChat were applied to deduce significant cell–cell communications. The resulting interaction networks were visualized using bubble plots for clarity. In this study, SPP1+ macrophages and tumor cells were set as receptors to calculate likely interacting ligands with the “netAnalysis_contribution” function, followed by “netVisual_circle” to trace the origin of these ligands, succinctly delineating the cellular communication landscape.

### Cell Culture and Reagents

The THP‐1, RBE cells, were purchased from Meisen Cell (Zhejiang, China). Cells were cultured in RPMI 1640 medium (Gibco, USA) with 10% fetal bovine serum (FBS) (Procell, Wuhan, China) at 37 °C in a humidified atmosphere with 5% CO2. For macrophage generation, 3 × 10^5 THP‐1 cells were seeded in six‐well dish treated with 100 ng PMA (MedChemExpress, USA) for 72 h and polarized into macrophages (THP1‐M0).

Macrophages (THP1‐M0) and RBE cell lines co‐cultivation was conducted using the non‐contact co‐culture transwell system (0.3 µm, Corning, USA). Place the pre‐cultured RBE cells in the upper chamber and combine it in a six‐well plate containing THP1‐M0. After that, seal the six‐well plate to create a limited oxygen environment. After 12 h of co‐culture, macrophages (THP1‐M0) were harvested for further analyses.

For tumor cell stimulation assays, human recombinant SPP1 protein (Cat: P02393, Solarbio, China) was added to RBE cells and cultured for 24 h in the presence or absence of fetal bovine serum. After that, cells were harvested for further analyses.

### Statistical Analysis

All data underwent suitable statistical evaluations. For variables exhibiting normal distribution, differences between two groups were assessed using an unpaired two‐tailed *t*‐test, while non‐normally distributed variables were compared with the Mann–Whitney U test. Multiple group comparisons, involving three or more groups, were conducted using the Tukey post‐hoc test. Survival differences were examined with the Kaplan–Meier method complemented by the log‐rank test. GraphPad Prism version 8.4.2 was utilized for scoring immunohistochemical results and for visualizing data. A *p*‐value of less than 0.05 was set as the threshold for statistical significance (*p < 0.05, **p < 0.01, ***p < 0.001).

## Conflict of Interest

The authors declare no conflict of interest.

## Author Contributions

L.Z., L.Z., and C.Q. contributed equally to the work. Z.J. and L.Z. conceived and designed the study. L.Z. analyzed the acquired data. Z.J., G.J., L.Z., and C.Q. supervised the integral analysis. Z.C., M.D., H.J., C.Z., Z.J., and L.Y. contributed to data collection, curation, and interpretation. All authors contributed to the manuscript and reviewed it carefully before submission.

## Code Availability

Codes used in this study are available at GitHub (https://github.com/TsuyinLee/Leading‐edge‐area‐in‐ICC).

## Supporting information



Supporting Information

Supporting Information

## Data Availability

The data that support the findings of this study are available from the corresponding author upon reasonable request.
