# Supplementary material for: Single‐Cell and Spatial Transcriptomics Delineate the Microstructure and Immune Landscape of Intrahepatic Cholangiocarcinoma in the Leading‐Edge Area
Source: Adv Sci (Weinh). 2024 Dec 24;12(7):2412740. doi: 10.1002/advs.202412740 (PMC11831447; doi:10.1002/advs.202412740)
Supplement: Supplementary file 1 — Supporting Information [file ADVS-12-2412740-s001.docx]

**Single-cell and Spatial Transcriptomics Delineate the Microstructure and Immune Landscape of Intrahepatic Cholangiocarcinoma in the Leading-edge Area**

Li Zuyin^1,2#^, Li Zhao^1,2#^, Cheng Qian^1,2#^, Zhang Changkun^1,2^, Ma Delin^1,2^, Hao Jialing^1,2^, Chen Zhuomiaoyu^1,2^, Li Yuzi^1,2^, Zheng Jiaxi^1,2^, Gao Jie^1,2*^, Zhu Jiye^1,2*^

1. Department of Hepatobiliary Surgery, Peking University Organ Transplantation Institute, Peking University People's Hospital, Beijing, China,100044

2. Beijing Key Surgical Basic Research Laboratory of Liver Cirrhosis and Liver Cancer, Beijing, China,100044

^#^These authors contributed equally to the work.

***Corresponding author:**

Zhu Jiye, Department of Hepatobiliary Surgery, Peking University Organ Transplantation Institute, Peking University People's Hospital; Beijing Key Surgical Basic Research Laboratory of Liver Cirrhosis and Liver Cancer, Beijing, China,100044. E-mail: zhu_jiye@163.com

Gao Jie, Department of Hepatobiliary Surgery, Peking University Organ Transplantation Institute, Peking University People's Hospital; Beijing Key Surgical Basic Research Laboratory of Liver Cirrhosis and Liver Cancer, Beijing, China,100044. E-mail: gaojie_1131@163.com


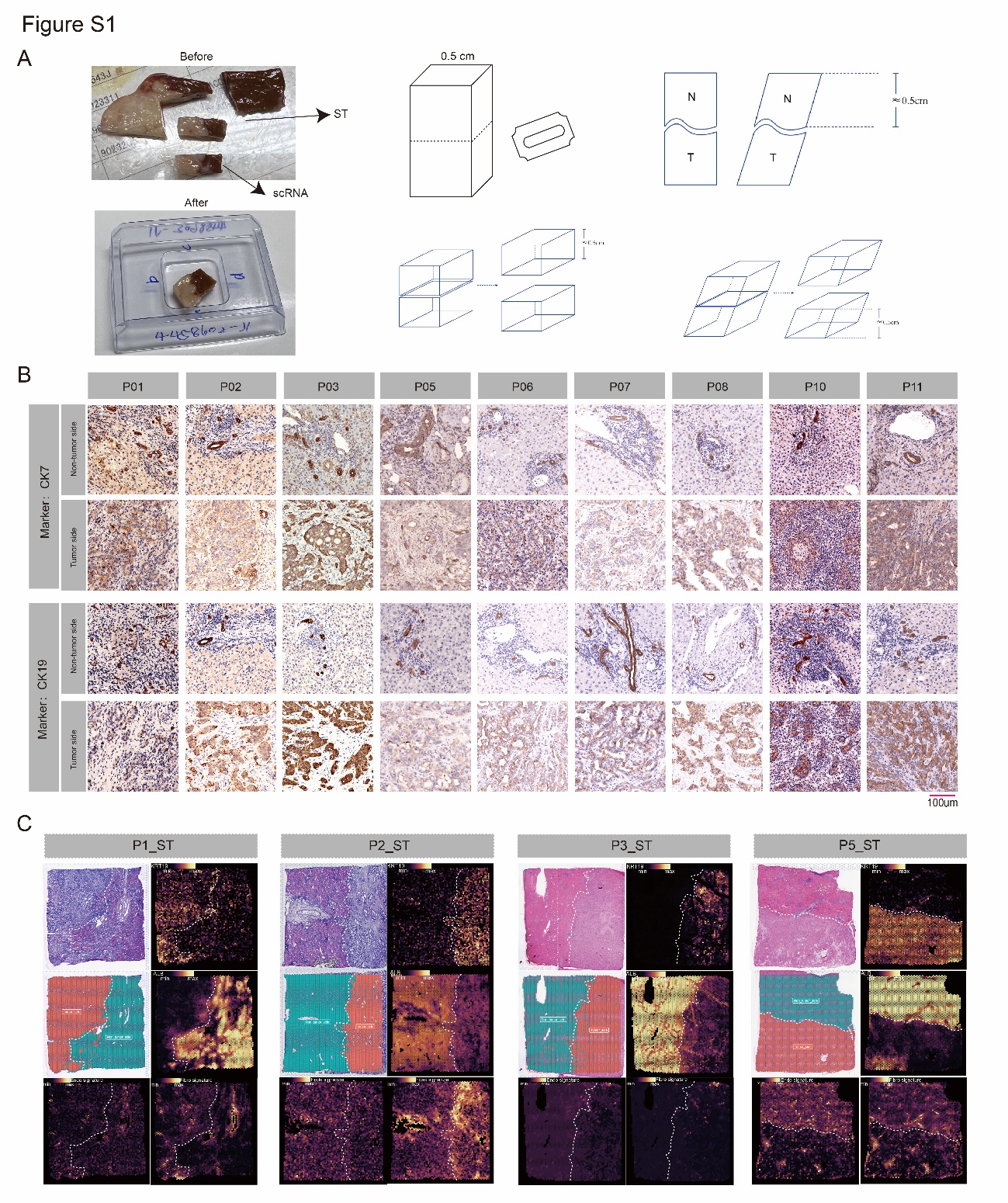


**Figure S1. The general outline of the leading-edge area depicted by single-cell and spatial transcriptome analysis.**

A) Schematic diagram of tissue processing in the lead-edge area. B) Representative H&E staining image of CK7 and CK19 in leading-edge area, including tumor side and non-tumor side within ICC samples. C) Spatial transcriptomic information of four other patients (P1, P2, P3, and P5)


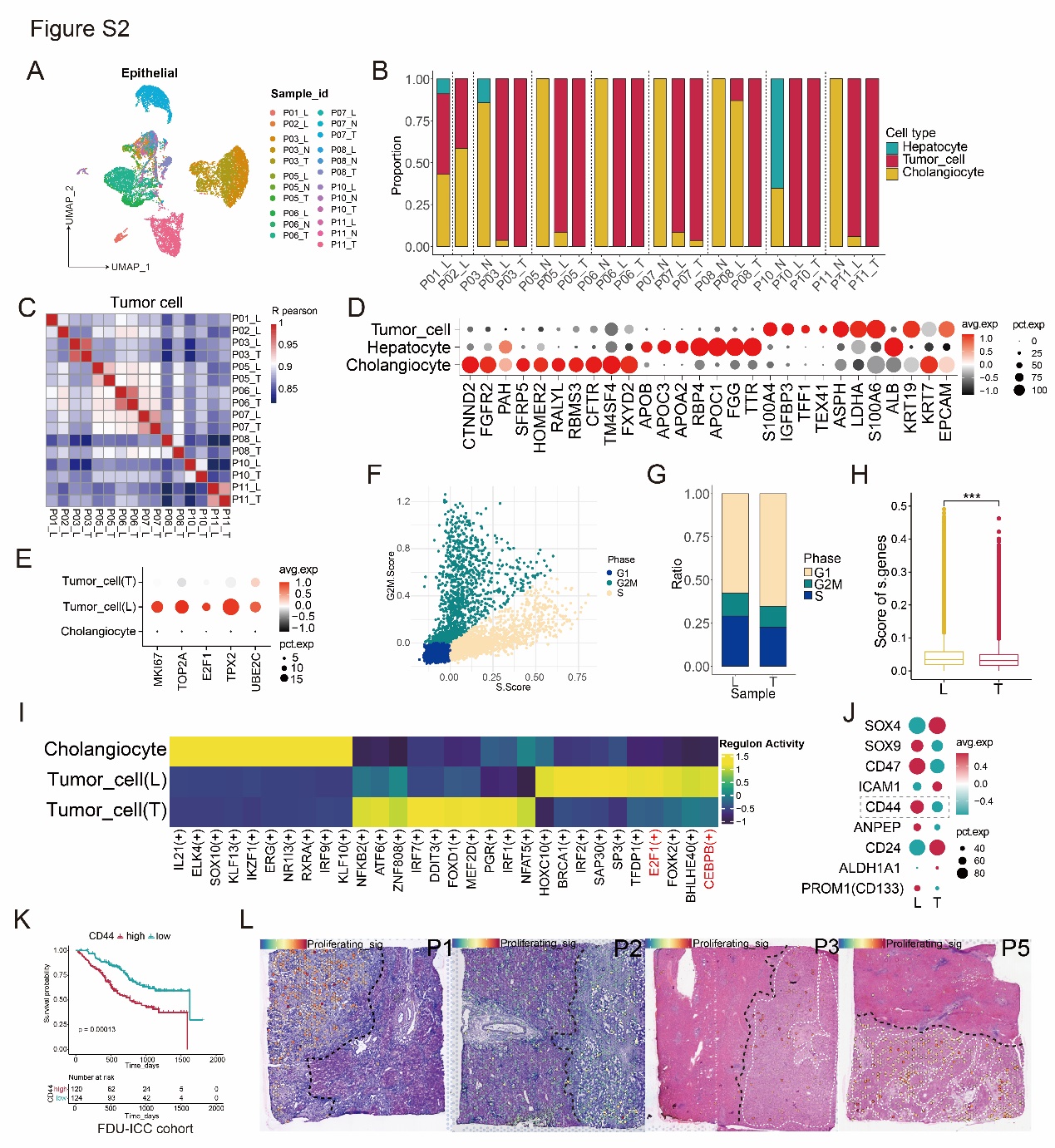


**Figure S2. ScRNA sequencing and spatial transcriptomics reveal ICC tumor cell heterogeneity at the leading-edge area**

A) UMAP plot shows the composition of epithelial from 23 ICC samples. B) The distribution of epithelial subpopulations across the analyzed samples. C) The genomic expression resemblance among various tumor cell samples. D) Circular plots illustrate the levels of distinct marker genes within diverse epithelial subsets. E) The dot plot shows the expression of proliferative marker genes in tumor cells in different regions (L- and T- side), with normal epithelium (cholangiocyte) as a reference. F-G) Identification of different cell phases of tumor cells (F) and their proportion (G) in two different regions. H) Comparison of S phase gene scores between two groups. I) A heatmap delineates the relative activity levels of the leading 10 TFs, as inferred by the pySCENIC method, across various epithelial subsets. J) Dot plot shows the expression of stemness markers in tumor cells between two regions. K) Kaplan-Meier survival curves were constructed to assess the correlation between CD44 mRNA expression and overall survival in the bulk RNA-seq dataset from Fudan University's ICC patient cohort. L) The spatial dot plots showed the distribution of proliferating tumor cells in four other patients. p < 0.05, **p < 0.01, ***p < 0.001. No significant difference (n.s.).


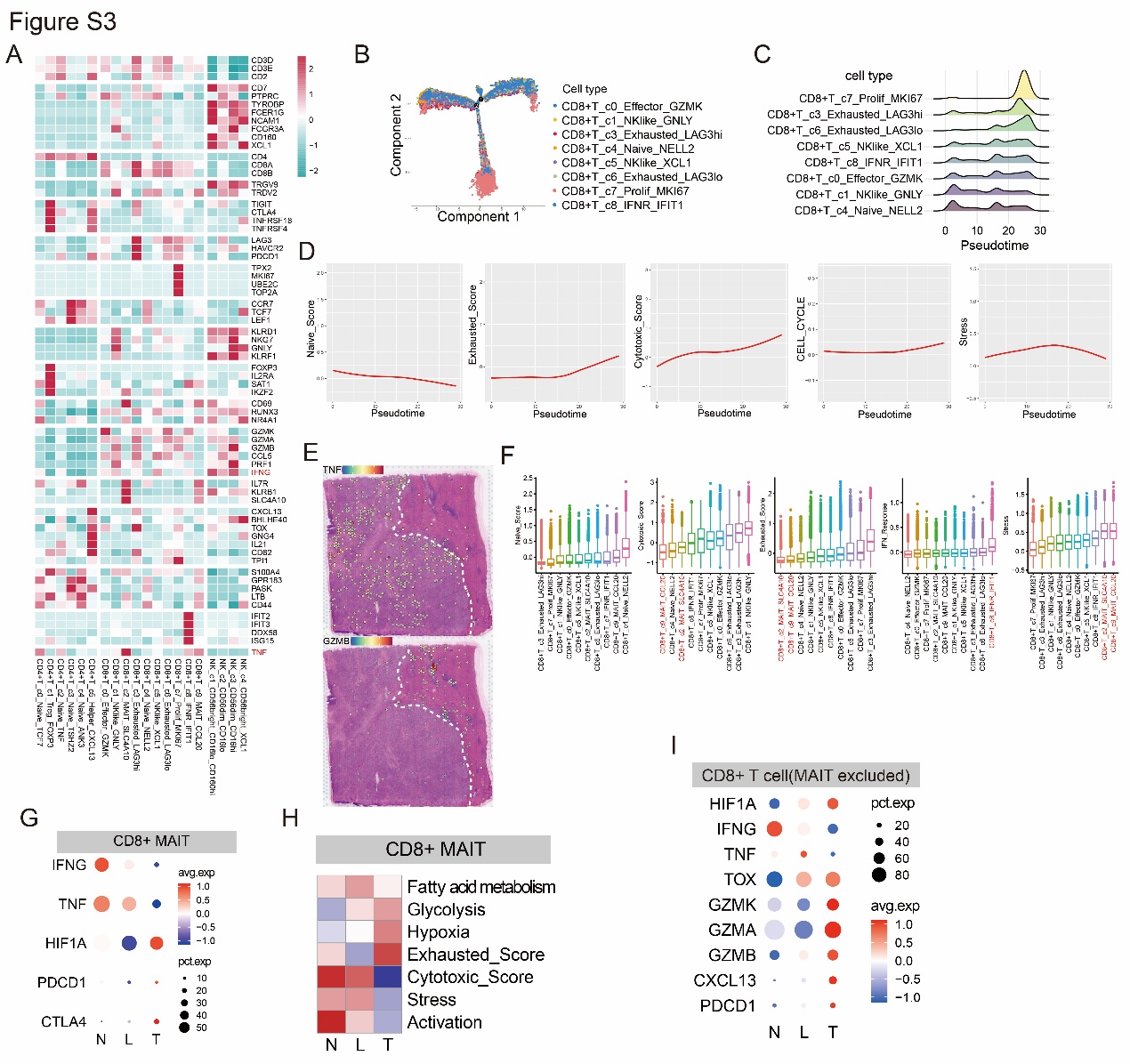


**Figure S3. Naive CD8+ T cells dominate and MAIT cells infiltrate in the leading-edge area.**

A) The heatmap shows the marker gene expression in each sub-cluster of T/NK cells. B-C) Pseudo-time trajectory analyzes the distribution of CD8+ T cell subsets across pseudo-time. D) Five different processes of CD8+ T cells change across pseudo-time. E) The spatial dot plots show the expression of the effector gene of MAIT(TNF) and other cytotoxic CD8+ T cells (GZMB). F) The scores of 5 different processes among CD8+T cell subsets. G) Dot plot shows the gene expression of MAIT in different groups, including effector markers, hypoxia markers, and immune checkpoint genes. H) The heatmap shows the comparison of different processes of MAIT cells among different groups. I) Dot plot highlights the gene levels of CD8+ T cells categorized by groups, featuring signatures for effector activity, hypoxia, exhaustion status, and immune checkpoint regulation.


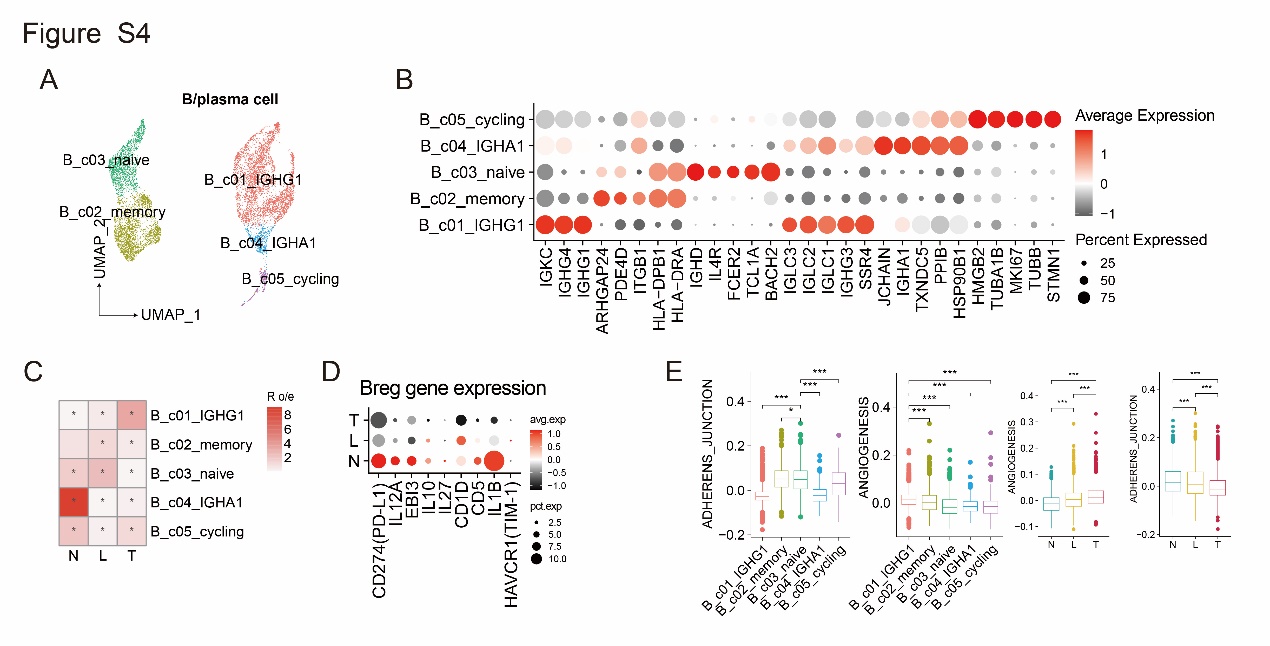


**Figure S4. Characteristics of B cell subsets in the leading-edge area**

A) UMAP plot shows the B cell subsets, colored by cell types. B) The diagram reveals the distinct marker gene expressions within various B cell subsets. C) The distribution tendency of B cell subpopulations in different groups. D) Dot plot shows the levels of the Breg genes in different groups. E) Comparison of two cellular processes of B cells in different cell types and positions. p < 0.05, **p < 0.01, ***p < 0.001. No significant difference (n.s.).


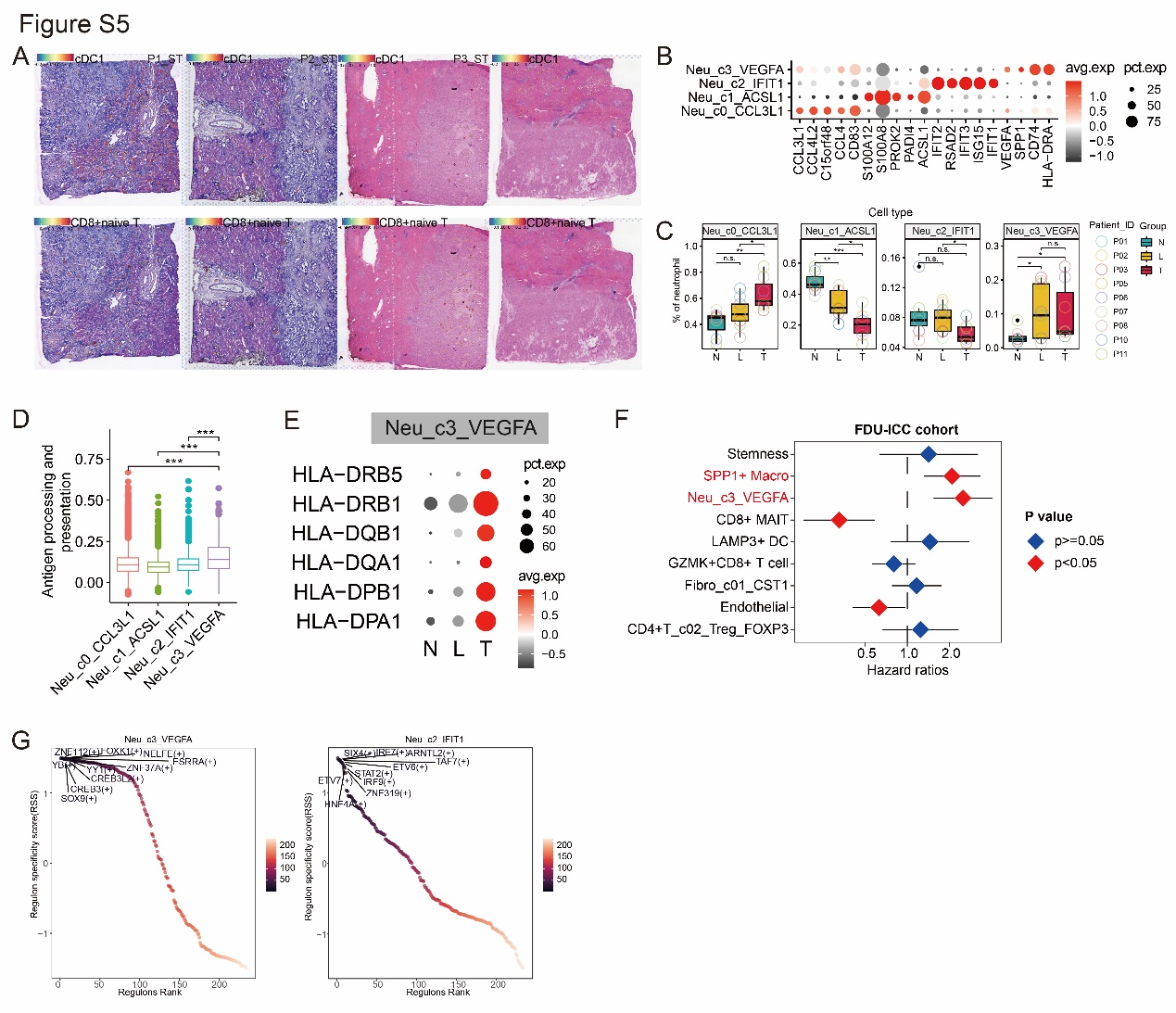


**Figure S5. The antigen-presenting capacity of APCs in the leading-edge area is impaired.**

A) The spatial dot plots show the distribution of cDC1, CD8+ naive T cells in the invasive tumor front of four other patients. B) The circular plot reveals the marker gene expression in each neutrophil subset. C) The proportion of neutrophil subpopulations at each location.

D) Comparison of antigen presentation and processing capacities among four subgroups of neutrophils E) The expression of MHC-II molecules of VEGFA+ neutrophil in each location. F) Univariate analysis of the impact of gene sets in each subpopulation on patient mortality. G) Top regulons identified with pySCENIC for the indicated neutrophil subtype cluster from the scRNA-seq data. p < 0.05, **p < 0.01, ***p < 0.001. No significant difference (n.s.).


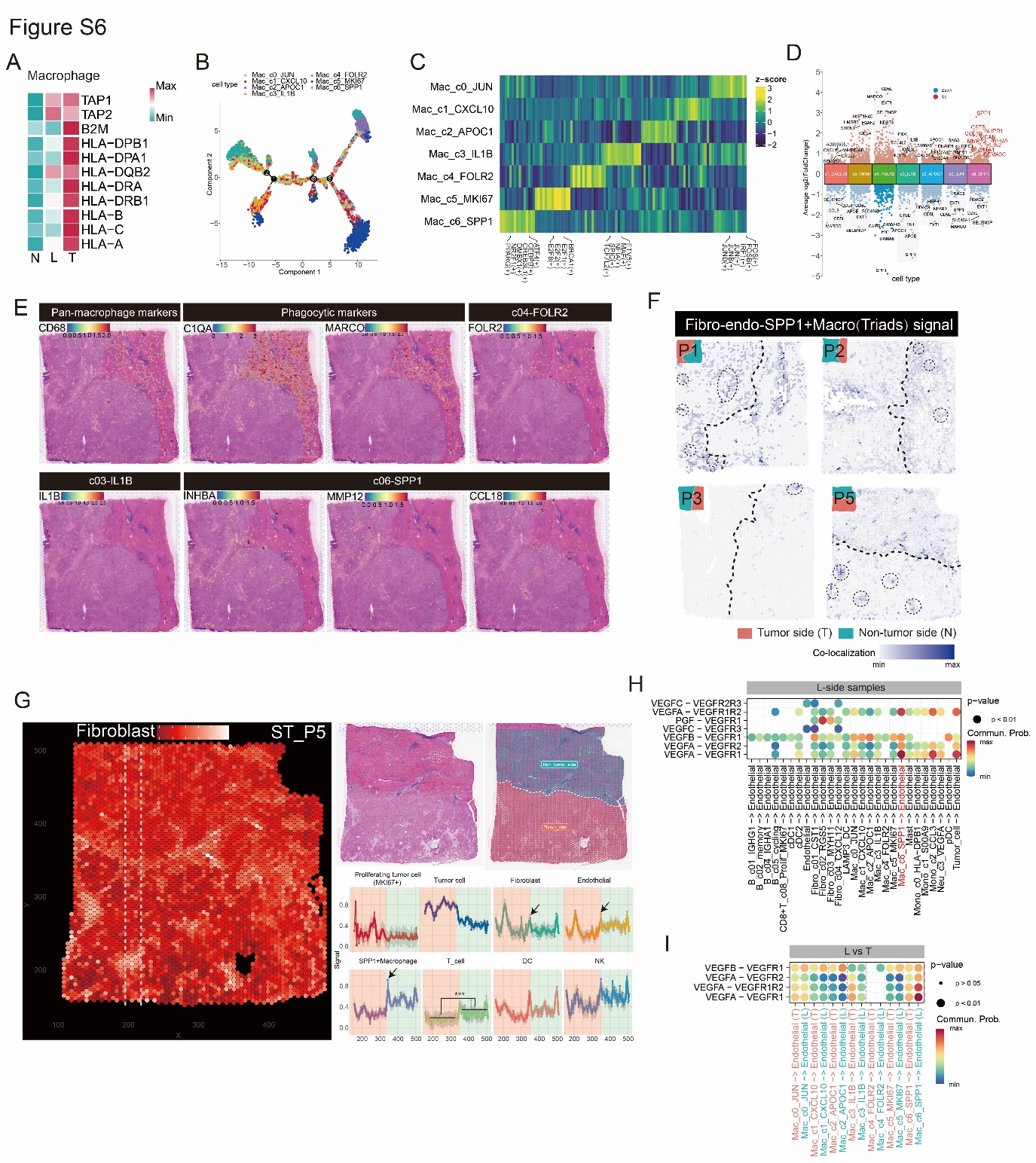
**Figure S6. Pro-angiogenic SPP1+ macrophages are the main macrophage subsets that infiltrate into the tumor side of the leading-edge area.**

A) The heatmap shows the levels of antigen presentation-related genes in macrophages at different regions. B) Potential developmental trajectory of macrophage subsets. C) The heatmap displays the top 10 transcriptional regulons of macrophage subpopulations. D-E) The markers of macrophage subsets (D) and the intensity and distribution of indicated markers in spatial sections (E). F) The distribution and intensity of ternary co-localization signals of fibroblasts, endothelial cells, and SPP1+ macrophages on four other spatial slices. G) The combinational graph shows the signature scores of fibroblasts, SPP1+ macrophages, T cells, and other indicated cells on the tumor side and non-tumor side, respectively. The signal is truncated from the mean signal within the dashed line. H) The selected ligand-receptor(L-R) interactions of all cell subsets to endothelial. Circle diameters represent P values, and the posibility of interaction is depicted through color gradation. I) Comparison of the interaction strength between SPP1+ macrophages in the core and frontier regions of tumors to endothelial cells through VEGFA-VEGFR1(L-R).


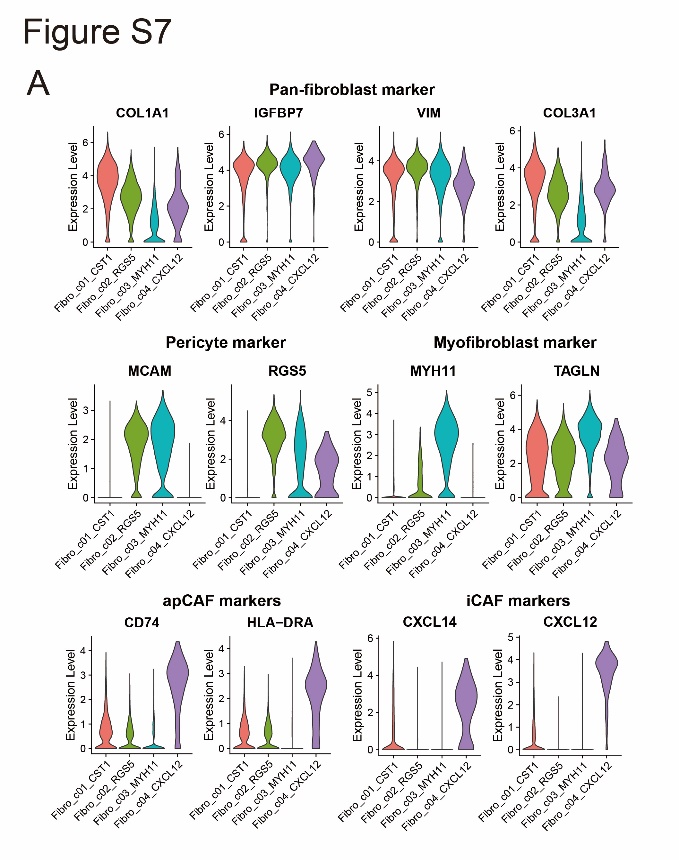


**Figure S7. POSTN+ FAP+ CAFs were the major fibroblast subset that composed that triad structure.**

A) Violin plots depict the expression profiles of canonical marker genes across the spectrum of fibroblast subpopulations, including pan-fibroblast, pericyte, myofibroblast, apCAF, and iCAF markers.


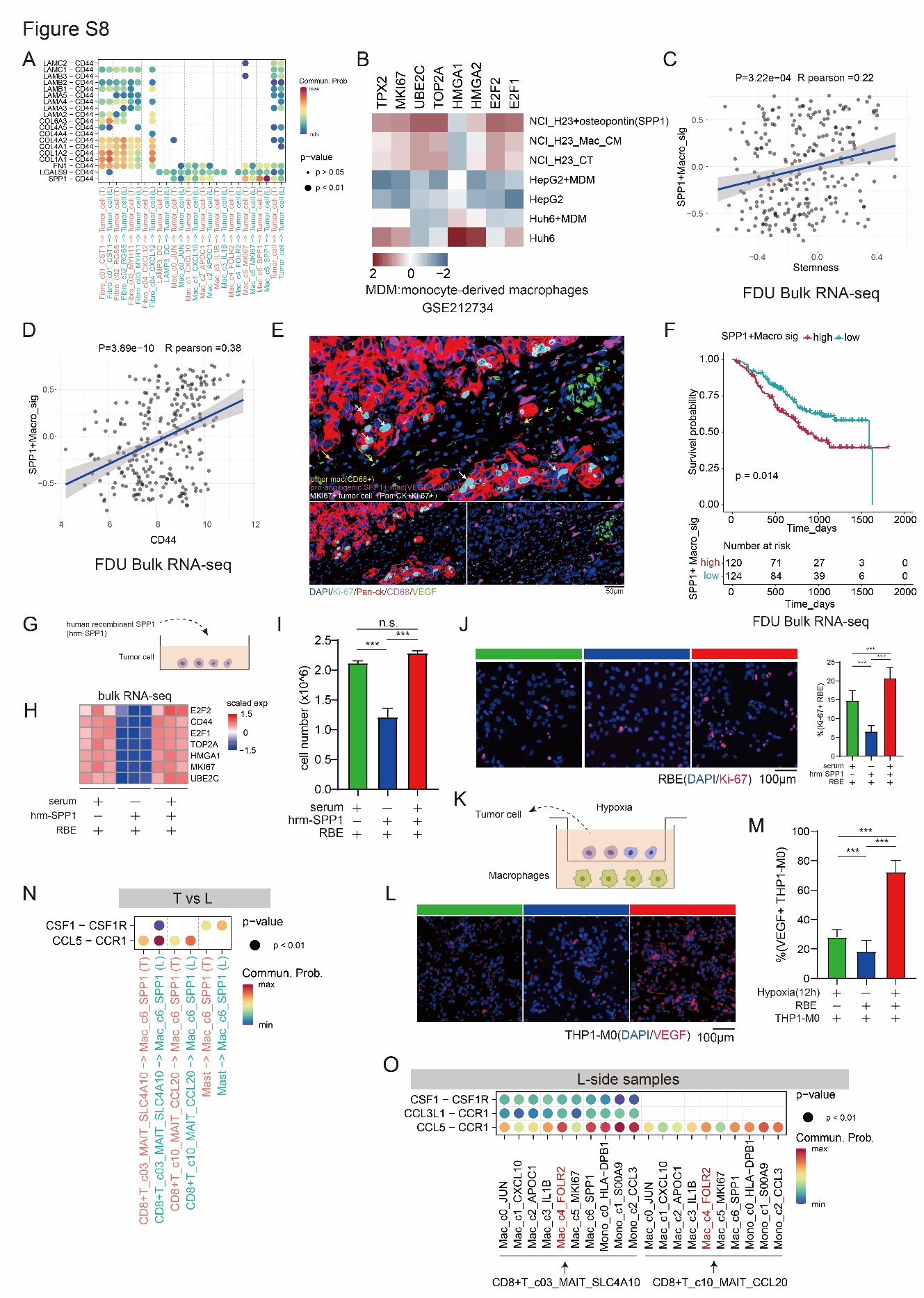


**Figure S8. Cellular crosstalk between tumor cells and immune cells shapes the unique TME of the invasive tumor front.**

A) The dot heatmap illustrates variances in intercellular signaling for specific ligand-receptor interactions, contrasting samples from the tumor core versus the leading-edge areas. B) The expression of proliferative markers in other tumor cells (Huh6, HepG2, and NCI-H23) co-cultured with MDM and SPP1 protein only. C) The association between the SPP1+ macrophage signature and tumor stemness signature within the Fudan University ICC bulk RNA sequencing dataset. D) Association analysis reveals the relationship between the SPP1+ macrophage signature and CD44 mRNA levels in the Fudan University (FDU) bulk RNA-seq ICC cohort. E) The mIHC images demonstrate the proximity of proliferating tumor cells (Ki-67+) to pro-angiogenic SPP1+ macrophages (VEGF+, CD68+) in the tumor side of the leading-edge sample. F) Kaplan-Meier survival curves were utilized to evaluate the prognostic significance of the SPP1+ macrophage signature on overall survival outcomes within the bulk RNA-seq dataset of Fudan University (FDU) ICC cohort. G) The schematic of RBE cell line stimulation with SPP1 protein, with or without fetal bovine serum. H-J) After the above treatments, the proliferation-related genes (H), tumor cell count (I), and the levels of Ki-67+ tumor cells in each group (J). K-M) Schematic of co-culture of tumor cells with macrophages (THP1-M0) under normoxic and hypoxic conditions (K), and the levels of VEGF+ THP1-M0 in each group (L-M). N) A dot heatmap delineates the variations in cell-to-cell signaling pathways from MAIT or MAST cells to SPP1+ macrophages, highlighting differences in specific L-R interactions across tumor-core versus leading-edge samples. O) A dot heatmap illustrates the chosen L-R engagements between CD8+ MAIT cells and monocyte-macrophage lineages. p < 0.05, **p < 0.01, ***p < 0.001. No significant difference (n.s.).


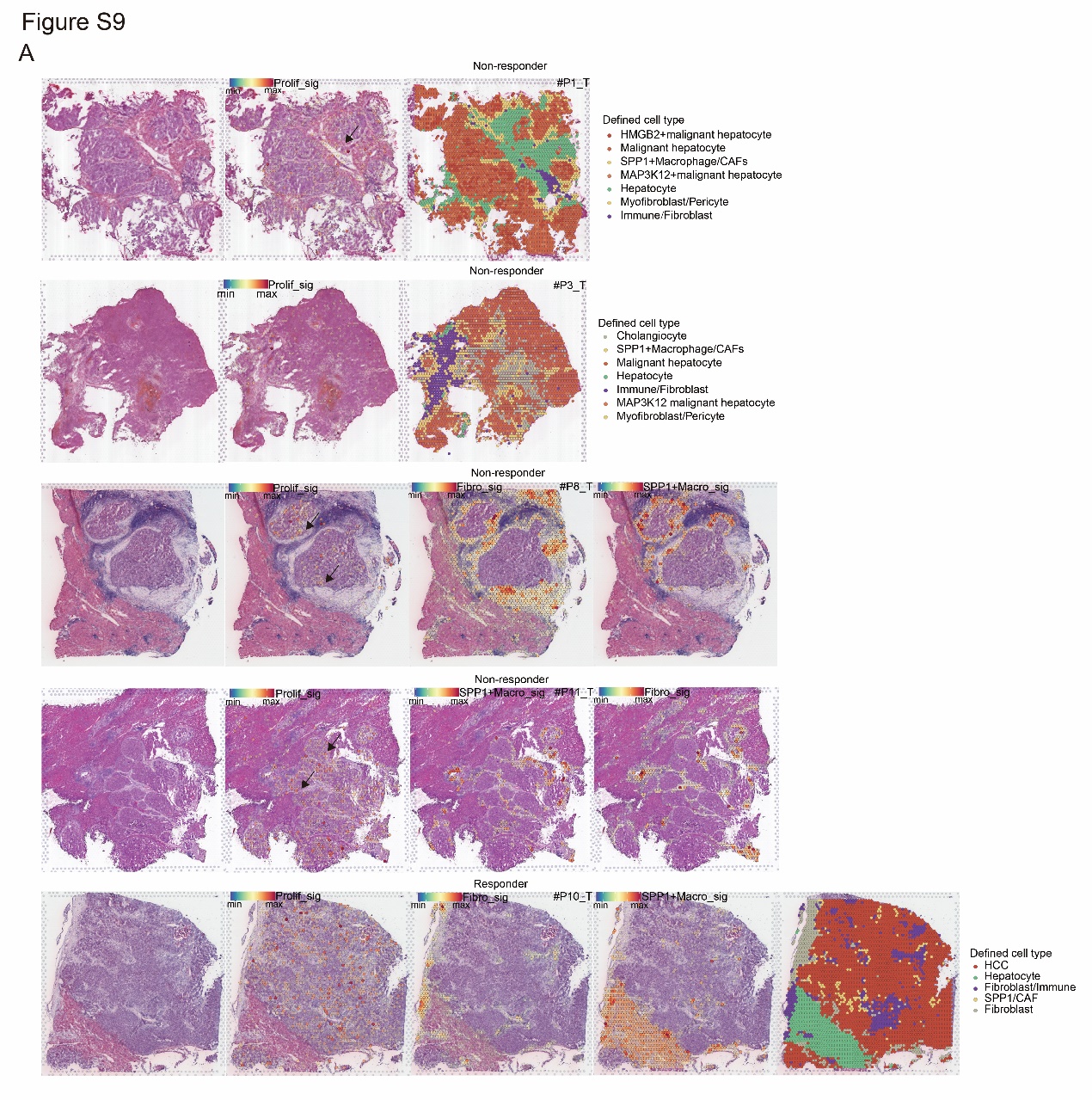


**Figure S9. The phenomenon of proliferative tumor cells being adjacent to the triad structure is also manifested in other types of tumors.**

A) In HCC, proliferative tumor cells are located near the stromal area composed of SPP1+ macrophages and fibroblasts.
